# Effects of Dietary Antimicrobial Growth Promoters on Performance Parameters and Abundance and Diversity of Broiler Chicken Gut Microbiome and Selection of Antibiotic Resistance Genes

**DOI:** 10.3389/fmicb.2022.905050

**Published:** 2022-06-16

**Authors:** Shyam Sundar Paul, Savaram Venkata Rama Rao, Nagendra Hegde, Nicola J. Williams, Rudra Nath Chatterjee, Mantena Venkata Lakshmi Narasimha Raju, Godumagadda Narender Reddy, Vikas Kumar, Prakki Santosh Phani Kumar, Sathi Mallick, Madhuranjana Gargi

**Affiliations:** ^1^Poultry Nutrition Lab, ICAR-Directorate of Poultry Research, Poultry Nutrition, Indian Council of Agricultural Research, Hyderabad, India; ^2^National Institute of Animal Biotechnology, Hyderabad, India; ^3^Department of Livestock and One Health, Institute of Infection, Veterinary and Ecological Sciences, University of Liverpool, Liverpool, United Kingdom; ^4^Director’s Lab, ICAR-Directorate of Poultry Research, Poultry Nutrition, Indian Council of Agricultural Research, Hyderabad, India

**Keywords:** amplicon sequencing, shotgun sequencing, chickens, gut microbiome, antimicrobial resistance, antibiotic growth promoter, broiler–chicken

## Abstract

Antimicrobial growth promoters (AGPs) are commonly used in broiler production. There is a huge societal concern around their use and their contribution to the proliferation of antimicrobial resistance (AMR) in food-producing animals and dissemination to humans or the environment. However, there is a paucity of comprehensive experimental data on their impact on poultry production and the AMR resistome. Here, we investigated the effect of five antimicrobial growth promoters (virginiamycin, chlortetracycline, bacitracin methyl disalicylate, lincomycin, and tylosin) used in the commercial broiler production in the Indian subcontinent and in the different parts of the world for three consecutive production cycles on performance variables and also the impact on gut bacteria, bacteriophage, and resistome profile using culture-independent approaches. There was no significant effect of AGPs on the cumulative growth or feed efficiency parameters at the end of the production cycles and cumulative mortality rates were also similar across groups. Many antibiotic resistance genes (ARGs) were ubiquitous in the chicken gut irrespective of AGP supplementation. In total, 62 ARGs from 15 antimicrobial classes were detected. Supplementation of AGPs influenced the selection of several classes of ARGs; however, this was not correlated necessarily with genes relevant to the AGP drug class; some AGPs favored the selection of ARGs related to antimicrobials not structurally related to the AGP. AGPs did not impact the gut bacterial community structure, including alpha or beta diversity significantly, with only 16–20 operational taxonomic units (OTUs) of bacteria being altered significantly. However, several AGPs significantly reduced the population density of some of the potential pathogenic genera of bacteria, such as *Escherichia coli*. Chlortetracycline increased the abundance of *Escherichia* phage, whereas other AGPs did not influence the abundance of bacteriophage significantly. Considering the evidence that AGPs used in poultry production can select for resistance to more than one class of antimicrobial resistance, and the fact that their effect on performance is not significant, their use needs to be reduced and there is a need to monitor the spread of ARGs in broiler chicken farms.

## Introduction

Chicken is the cornerstone of animal agriculture worldwide and represents one of the most efficient forms of animal protein production with highly efficient feed conversion. Sustainable poultry meat and egg production are important to provide safe and high-quality protein sources for human nutrition.

The gastrointestinal tract of chickens is densely populated with diverse and complex microbiota (bacteria, fungi, archaea, protozoa, and viruses; dominated by bacteria), that play a vital role in the digestion and absorption of nutrients, host immune system development and pathogen exclusion, endocrine activity, maintenance of normal physiological homeostasis, and influencing the gut development, nutrient supply, and host metabolism and detoxification (Sommer and Bäckhed, 2013; Paul et al., 2021a). Antibiotics have been used as growth promoters in poultry production to prevent diseases and to improve growth, gut health, and feed efficiency for many years. It is thought that antimicrobial growth promoters (AGPs) alter the gut microbial community composition by inducing populations favorable to growth, immunity, and gut health. The use of AGPs, wherein antimicrobials are used at sub-therapeutic doses for a longer duration, favors the selection and spread of resistant genes of bacteria within animals and in humans through the food chain or other environmental pathways (da Costa et al., 2013). It has been emphasized that there are no geographic boundaries to contain the spread of antimicrobial resistance and if preventive and containment measures are not applied locally, nationally, and regionally, the limited interventions in one country, continent, and for instance, in the developing world, could compromise the efficacy and endanger antimicrobial resistance containment policies implemented in other parts of the world, including the best-managed high-resource countries (Founou et al., 2016). Considering the gravity of the potential threat to human and animal health, some countries have banned the use of AGPs, while many countries including India are still using various antimicrobials, such as AGPs. Although different alternatives to antibiotic growth promoters are now available in the market, AGPs are still commonly being used by farmers due to their easy availability and their cheaper cost than that of the alternatives as farmers commonly believe that AGPs may prevent common pathogens from being established in the gut at low cost. The use of a particular antimicrobial may induce co-resistance to other antimicrobials that were not administered, primarily due to the co-selection of linked genes encoding otherwise unrelated resistance mechanisms to different antimicrobials (Threlfall, 2000; Ciric et al., 2011).

There is a paucity of data about the effects of sub-therapeutic doses of antimicrobials on shifts in the gut microbial community structure or density of various ARGs. A limited effort has been made to evaluate the comparative effectiveness of commonly used AGPs in modulating gut microbiota of chickens favorably under controlled scientific feeding trials. Further, understanding the effects of AGPs on the diversity and community structure of the gut microbiome is important for devising strategies for developing alternatives to AGPs for improving the chicken gut microbiome. In addition, little or no information is available on the effects of AGPs on gut bacteriophages (viruses of gut bacteria) which are known to shape the gut bacterial composition and facilitate horizontal gene transfer (Sutton and Hill, 2019), and whether they influence the abundance of antimicrobial resistance genes (ARGs) in gut microbial communities. Further, limited information is available on the influence of one class of AGP on the development of resistance for other classes of antimicrobials. It is also important to assess if commonly used AGPs favor the accumulation of ARGs related to critically important therapeutic antibiotics for human or animal health.

There have been various approaches for investigating the gut microbiome and resistomes. Valuable information has been generated by culture-based approaches; however, culture-independent methods utilizing shotgun sequencing of metagenomic DNA extracted from gut contents have the advantage of detecting and quantifying culturable as well as unculturable microbes. In addition, they provide information on the whole ARG complement; therefore this approach provide a global view of both the microbial community and its resistome profile. Although both the 16S rRNA gene amplicon and shotgun sequencing methods allow for the profiling of microbiome, many researchers have demonstrated that shotgun sequencing allows for enhanced detection of bacterial species as compared to amplicon sequencing (Jovel et al., 2016; Ranjan et al., 2016). However, a large number of studies reported that amplicon sequencing detects significantly more phylum and family level diversity (Tessler et al., 2017). Microbiome datasets generated by high throughput sequencing (amplicon or shotgun) are compositional because they have an arbitrary total imposed by the capacity of the instrument and the count cannot be related to the absolute number of molecules in the input sample (Gloor et al., 2017). Quantitative real-time PCR (qPCR) is one of the most widely used methods to precisely quantify bacteria or genes in a complex ecosystem. Hence, it is expected that employing these three techniques together may complement each other.

In the present study, we examined the influence of five AGPs commonly used in the commercial broiler production in the Indian subcontinent on the performance parameters and community composition of bacteria and bacteriophages and the abundance and selection of genes related to antimicrobial resistance using 16S amplicon and shotgun sequencing and qPCR assays of chicken hindgut metagenomic DNA.

## Materials and Methods

### Animals, Treatments, and Experimental Design

Three experimental feeding trials were conducted consecutively with commercial broiler strain (Vencobb 430) to evaluate the effect of five antimicrobial growth promoters (AGPs) along with one negative control (C) group. Air-dried litter of the preceding production cycle (trial) was offered groupwise on chicks of the next cycle for 10 days, besides a balanced diet, to simulate carry-over effects of ingestion of built-up litters, as is expected in a deep litter system of rearing broilers.

A total of 360, 390, and 420 newly hatched chicks obtained from a local hatchery (Venkateshwara Hatcheries Pvt Ltd., Hyderabad, India) were randomly divided into 72, 78, and 84 pens (clean stainless steel battery brooder cages) containing 5 birds in each pen measuring 6 ft^2^, respectively during the first, second, and third cycles. Twelve, thirteen, and fourteen replicate pens were assigned to each of the 6 treatments, respectively, during the first, second, and third cycles in a completely randomized design. All the groups received the same basal diet. The negative C group was not supplemented with any AGP. Five other groups were supplemented with one of the five AGPs namely virginiamycin (V or VIR, 40 g/t feed), chlortetracycline (CT or CTC, 330 g/t feed), bacitracin methylene salicylate (B or BMD, 500 g/t feed), lincomycin (L or LIN, 40 g/t feed), and tylosin (T or TYL, 500 g/t feed) at dose levels recommended by respective manufacturers.

During all the three cycles/trials, all the chicken were fed with the same maize and soybean-based balanced diet *ad libitum* as per feeding standards for an intensive production system. The detail of diet composition has been presented in Supplementary Table 1. The Institute of Animal Ethics Committee of ICAR-DPR approved the experiments (approval number IAEC/DPR/18/10) and the methods were performed as per the guidelines of animal experimentation. All the birds were housed in an open-sided poultry house. The average minimum and maximum temperatures in the house during the experiments range from 23.5 to 37.5, 23.1 to 37.1, and 25.9 to 35.5*^o^*C, respectively, during the three cycles. Birds were wing-tagged, weighed, and vaccinated with Marek’s disease vaccine on arrival. Brooding was done with the help of incandescent bulbs for up to 21 days. The feed was offered and made available in the feeders placed in each pen to ensure the constant availability of feed for all the birds throughout the period. *Ad libitum* access to the drinking water was provided through separate drinkers in each pen. Birds were vaccinated with the New castle disease Lasota strain vaccine on 5th and 28th days and with infectious bursal disease vaccine on the 10th and 16th days. Weekly body weight (BW) was recorded for each pen and per bird average weight was calculated for each pen. A weighed quantity of feed was offered daily, and the leftover feed was weighted at weekly intervals. Mortality was recorded daily, and the pen number, the wing band number, and BW of the dead birds were recorded. The feed conversion ratio was adjusted for mortality (Paul et al., 2021b). Cages were placed distantly so that there was no fecal contamination among pens.

### Metabolism Trial

A metabolism trial was conducted during cycle 1 on birds in four replicate pens from each group (20 birds per group) during the 4th week of age using the total collection method. Briefly, total excreta were collected, weighed, and dry matter (DM) content was estimated in each pen for 3 consecutive days, and the samples were pooled penwise for chemical analysis. Daily feed intake in each pen was also measured. Proximate composition nitrogen, organic matter (OM), and ether extract (EE) in feed and excreta were estimated as per procedures described by the Association of Official Analytical Collaboration AOAC (1990). The nitrogen retention and apparent digestibility coefficients of DM, OM, and EE of the feed were calculated according to the standard formulae for the total collection method (Maynard and Loosli, 1969).

### Carcass Traits and Gut Content Sample Collection

From each group, ten apparently healthy chickens in their finishing (marketable age) stage (at the 36th day) were selected (one per pen), caught, and euthanized by cervical dislocation. The carcass weight, breast muscle, abdominal fat pad, the heart, liver, spleen, bursa, and gizzard weight were recorded, and their weights were calculated relative to their live body weight.

The gut was opened immediately using sterile scissors and luminal contents of the hindgut (from the duodenum to cloaca including ceca) were recovered into sterile cryovials. For every 1 g gut content, 5 ml of 1X phosphate buffer saline was added and mixed thoroughly by vortexing to produce a uniform homogenate. If necessary, a sterile 1 ml pipette tip was used to break up any large particulate matter that may remain in suspension while vortexing. The homogenated gut content was immediately stored in a portable freezer at –20°C, transported to the laboratory, and stored at –80°C.

### DNA Extraction

Metagenomic DNA was extracted from the pooled hindgut contents of individual chicken following the repeated bead beating plus column method described by Yu and Morrison (2004) using the DNA purification columns from the commercially available QIAamp Fast DNA Stool Mini kit (QIAGEN, Germany). DNA concentration and quality were assessed using a Qubit 3.0 fluorometer (Thermo Fisher Scientific, MA, United States; #Q33238) using DNA HS Assay Kit (Thermo Fisher Scientific, MA, United States; #Q32851) and gel electrophoresis. The DNA was stored at –20°C until further processing.

### 16S rRNA Gene Amplicon Sequencing, Sequence Processing, and Analysis

Hindgut microbiota from experimental cycle 1 was profiled by sequencing the v3–v4 region of the 16S rRNA gene for 18 DNA samples (from 3 birds slaughtered at 36 days of age, randomly taken from separate pens for each of the 6 groups). The extracted DNA was amplified using the primer pair, S-D-Bact-0342-b-S-17 (5′-CCTACGGGNGGCWGCAG-3′) and S-D-Bact-0785-1-A-21 (5′-GACTACHVGGGTATCTAATCC-3′) recommended by Klindworth et al. (2012) as per the protocol described earlier (Paul et al., 2021a). The amplified product was checked on 2% of agarose gel and gel purification was done to remove non-specific amplifications. In total, 5 ng of amplified product was used for library preparation using the NEBNext Ultra DNA library preparation kit (New England Biolabs, Ipswich, MA, United States). The library quantification and quality estimation were done in an Agilent 2200 Tape Station. Sequencing was performed using an Illumina HiSeq 2500 sequencer in 2 × 250 bp pair-end sequencing mode. Samples were processed with three negative controls per plate in the sequencing run. The raw reads obtained after demultiplexing were subjected to quality screening using FastQC (version 0.11.8) with default parameters. Primers and adapters were removed using in-house Perl scripts and reads with a Phred quality score (*Q* > 20) were considered for consensus sequence generation. The reads were merged using FLASH (Tanja and Salzberg, 2011) program (version 1.2.11) and consensus reads with an average contig length of 350–450 bp were obtained. Chimeras were removed using the *de novo* chimera removal method, UCHIME (version 11) as implemented in VSEARCH (Rognes et al., 2016). The operational taxonomic units (OTUs) picking and taxonomic assignments were performed using the quality-passed consensus v3-v4 sequences using UCLUST program as implemented in the bioinformatics software suite, “Quantitative Insights into Microbial Ecology” (QIIME) (Caporaso et al., 2010a) (version 2) software using a similarity cutoff of 0.97. A total of 1,68,910 OTUs with less than 5 reads were removed and the remaining 12,210 OTUs were further used for analysis. The representative sequences from each clustered OTUS were aligned against the SILVA core set of sequences using the PyNAST program (Caporaso et al., 2010b). Taxonomic classification was performed using the RDP classifier (Cole et al., 2014) by mapping each representative sequence against the SILVA database (138.1, 2020) (Quast et al., 2013). The BIOM file obtained from QIIME2 along with the sample metadata files were uploaded to the METAGENassist website (Arndt et al., 2012), where samples were normalized using the total sum (sample vs. sample) and Pareto scaling (taxon vs. taxon; mean centered and divided by the square root of the standard deviation of each variable). The processed data were used for the generation of taxonomic bar plots. The BIOM file along with the metadata file was also uploaded to MicrobiomeAnalyst (Dhariwal et al., 2017) for the analysis of alpha diversity, beta diversity, rarefaction curve, and differential abundance analysis. For the analysis of alpha diversity, beta diversity and rarefaction curve data were rarefied to the minimum library size (at 66,694 sequences per sample). For analysis of differential abundance, the data were normalized using the cumulative sum square scaling method.

### Shotgun Metagenomic Sequencing, Sequence Processing, and Analysis

For shotgun metagenomic sequencing, 24 DNA samples (4 individual birds slaughtered at 36 days of age were sampled per group; each bird from a separate pen) from the third cycle were selected randomly. QC-passed DNA samples (100 ng DNA per sample) were fragmented and tagged with sequencing adapters using the TruSeq Nano Library preparation kit (Illumina, San Diego, CA, United States; #20015964), and the extracted DNA samples resulted in libraries of the appropriate size and concentration to be sequenced. Shotgun metagenomic sequencing was performed using the NovaSeq 6000 sequencer (Illumina) at 150 bp in paired-end mode.

Following sequencing, all reads were assessed for quality parameters and were subjected to trimming, adapter removal, host genome sequence removal (using Bowtie (Langmead and Salzberg, 2012), SAMtools (Danecek et al., 2021), and BEDTools (Aaron et al., 2010) against *Gallus* (taxonomy is 9031)) and pair-end read merging using fastp (version 0.23.1) program (Chen et al., 2018). The Kaiju webserver was used in default mode for the taxonomic assignment of sequences. Kaiju translates reads into amino acid sequences and then compares them to a reference database containing bacterial, fungal, viral, and microbial eukaryotic protein sequences using the Burrows–Wheeler algorithm and assigns each read to a taxon in the NCBI taxonomy. By using protein level classification, Kaiju has been shown to achieve a higher sensitivity compared with methods based on nucleotide comparison (Menzel et al., 2016). Kaiju output was converted to OTU table and taxonomy table using a python script^1^.

The OTU table and consensus taxonomy files along with the metadata file were converted to phyloseq object and analyzed using R (R Core Team., 2020). OTUs classified as taxons other than bacteria were removed. For alpha diversity and rarefaction analysis, the data were rarified to even depth (at 7607352 sequences per sample). For the analyses of differential abundance (using DESeq2), the creation of stacked bar plots and upset diagram data were filtered for the low count and low variance OTUs (OTUs with <5 members or appearing in <2 samples were removed; this process removed 14,893 OTUs out of 3,3891 bacterial OTUs), OTUs with ambiguous phylum level annotation were also removed (this process removed 835 OTUs) to focus on important features and to improve the downstream statistical analysis, and then data were normalized using cumulative sum scaling (CSS) method. Taxonomic assignments (for the top 15 taxa) were presented as stacked bar plots from CSS normalized relative percent abundance data.

Beta diversity profiling and significance testing were carried out at the OTU level using principal coordinate analysis (PCoA) as well as non-metric multidimensional scaling (NMDS) ordination based on different distance methods, such as Bray–Curtis dissimilarities, Jaccard index, and Jensen–Shannon diversion, using statistical methods, such as permutational multivariate analysis of variance (PERMANOVA) and homogeneity of group dispersion (PERMDISP) using the MicrobiomeAnalyst web server after disabling default settings for data filtering for the low count and low variances but data were rarefied to minimum library size before analysis.

For bacteriophage detection, sequences were analyzed using FastViromeExplorer (Tithi et al., 2018).

For the estimation of the abundance of AMR genes, Groot, a tool to profile antimicrobial resistance genes in metagenome (resistome profiler software) and the “groot-db” a preclustered database derived from ResFinder, ARG-annot, and CARD databases provided with the software were used (Rowe and Winn, 2018).

Bubble plots were created using R. All data presented in Figures 6, 7 are untransformed but the data are normalized (normalized to per million reads)

### PCR and Quantitative Real-Time PCR Analyses

#### Quantification of Population Sizes of *Escherichia coli* and *Lactobacillus* spp.

The population sizes of *E. coli* and *Lactobacillus* spp. were quantified (in 6 replicate samples per group in the first as well as third cycle) using Maxima SYBR-Green based qPCR master mix (Genetix Biotech Asia Pvt Ltd., New Delhi, India), respective specific primers (Hermann-Bank et al., 2013; Torok et al., 2013), and an ABI StepOne quantitative PCR instrument (Thermo Fisher Scientific, MA, United States). The specificity of the primers was checked *in silico* against RefNR sequence collection of silva SSU r 138.1 database using the tool, TestPrime available in the SILVA website^2^. The qPCR reaction was performed in triplicate, along with no-template controls as per the master-mix manufacturer’s instruction. To minimize the potential bias, instead of a single strain, sample-derived qPCR standards were prepared using the respective specific PCR primer set and a composite metagenomic DNA sample that was prepared by pooling equal amounts of all the metagenomic DNA samples, and a standard curve was developed as described earlier (Stiverson et al., 2011). The absolute abundances were expressed as the number of *rrs* gene copies/50 ng DNA samples.

#### Screening of Antimicrobial Resistance Genes Using Conventional PCR

For the screening of AMR genes using conventional PCR, 6 samples per group from the first as well as the third cycle were utilized. A detailed list of primers and protocols used for the detection of some of the important and commonly found AMR genes in hindgut metagenomic DNA samples is presented in Supplementary Data Sheet 1. Briefly, *bla* genes were screened using multiplex PCR as per Dallenne et al. (2010). The *bla*_*CTX–M*_ genes were screened initially using universal primers as per Boyd et al. (2004) followed by the analysis of positive samples using multiplex PCR involving primers for different subgroups as per Dallenne et al. (2010). Quinolone resistance genes were screened using multiplex PCR as per Ciesielczuk et al. (2013). Colistin resistance genes (mcr 1 to 4) were screened using multiplex PCR as per Lescat et al. (2018).

#### Quantitative Real-Time PCR Based Quantification of Antimicrobial Resistance Genes

All the samples that tested positive for any of the above-mentioned AMR genes by conventional PCR were further analyzed for copy number estimation using qPCR using primers and protocols as listed in Supplementary Data Sheet 1.

### Statistical Analysis

The data on performance variables, slaughter data, digestibility, and qPCR data on *E. coli* and *Lactobacillus* spp. were analyzed by one-way analysis of variance (ANOVA) using the general linear model univariate procedure available in SPSS (2008). At the detection of overall significant difference, specific differences between pairs of means were tested using Duncan’s multiple range test at *P* < 0.05.

For both the 16S amplicon sequencing and shotgun sequence data, at the detection of significant difference in the overall abundance between groups on DESeq2 analysis, followed by Benjamini–Hochberg false discovery rate (FDR), correction of *p*-Values for multiple comparisons, groups were compared pairwise using non-parametric Mann–Whitney *U*-test (Wilcoxon rank-sum test) as implemented in SPSS (2008). Alpha diversity matrices were compared at the OTU level using the non-parametric Wilcoxon test, as implemented in the MicrobiotaProcess library^3^ in R. Beta diversity profiling and significance testing were carried out using NMDS as well as PCoA ordination based on different distance methods, using statistical methods, such as PERMANOVA and homogeneity of group dispersion of permutational analysis of multivariate dispersions (PERMDISPs).

Data on bacteriophage and AMR genes were normalized to per million reads and tested for normality (Shapiro–Wilk test and univariate normal plots) and equal variance (Levane’s test). For statistical analysis, normalized data on bacteriophage and AMR genes were transformed to the square root to fulfill the equal variance condition of the ANOVA test. These data were statistically analyzed using an ANOVA test followed by Duncan’s multiple range test as implemented in SPSS.

## Results

### Effects on the Performance Parameters and Digestibility

During Cycle 1, body weight gain (BWG) was significantly higher in some of the groups with AGPs during the first 21 days, but by the end of the trial (at 35th day), there was no significant effect of AGPs on the growth performance or feed efficiency (Table 1). During cycle 2 as well as cycle 3, there was no significant effect of AGPs on the growth performance or feed efficiency in any of the phases.

**TABLE 1:** Effect of supplementing different antibiotic growth promoters on the performance of broiler chicken during three consecutive production cycles.

	C	V	CT	B	L	T	SEM	N	*P*-value
Performance during cycle 1									
1-14*^d^*									
BWG	433.6	463.3	440.2	446.9	449.3	455.3	3.126	12	0.091
FE	1.106	1.086	1.112	1.095	1.129	1.126	0.008	12	0.616
1-21*^d^*									
BWG	774.0*^c^*	868.0*^a^*	836.3*^ab^*	808.3bc	828.7*^ab^*	867.8*^a^*	7.211	12	0.001
FE	1.193	1.165	1.202	1.213	1.239	1.214	0.007	12	0.070
1-35*^d^*									
BWG	2007	2046	1994	2022	2013	2040	12.50	12	0.843
FE	1.342	1.387	1.379	1.374	1.371	1.383	0.010	12	0.837
Performance during cycle 2									
1-14*^d^*									
BWG	412.0	434.2	418.2	434.8	427.2	419.0	3.99	13	0.489
FE	1.190	1.169	1.191	1.179	1.193	1.211	0.004	13	0.064
1-21*^d^*									
BWG	854.2	880.6	856.2	860.4	878.6	825.5	7.826	13	0.368
FE	1.282	1.274	1.297	1.290	1.285	1.314	0.006	13	0.503
1-35*^d^*									
BWG	2020	2016	2013	1960	1986	1931	18.76	13	0.701
FE	1.465	1.490	1.523	1.516	1.489	1.527	0.010	13	0.494
Performance during cycle 3									
1-14*^d^*									
BWG	379.1	392.1	377.8	385.5	386.2	362.2	3.373	14	0.163
FE	1.279	1.273	1.280	1.269	1.293	1.280	0.004	14	0.671
1-21*^d^*									
BWG	792.6	829.9	795.3	810.7	806.0	767.3	6.637	14	0.137
FE	1.385	1.366	1.375	1.393	1.397	1.393	0.005	14	0.489
1-35*^d^*									
BWG	1842	1807	1834	1876	1833	1798	14.34	14	0.691
FE	1.622	1.679	1.604	1.617	1.614	1.601	0.013	14	0.570

*V, virginiamycin (40 g/ton); CT, chlortetracycline(330 g/ton); B, bacitracin methylene disalicylate (500 g/ton); L, lincomycin (40 g/ton); T, tylosin (500 g/ton); BWG, body weight gain; FE, body weight gain/feed intake; P, probability, N, number of replicate pens; SEM, standard error of the mean; Means having common superscripts in a row do not vary significantly (P < 0.05).*

There was no significant difference in the slaughter parameters (carcass weight, breast weight, the heart, liver, and abdominal fat weights) among the groups (Supplementary Table 2).

Overall, there were 4.16, 2.46, 2.38, 4.2, 1.97, and 3.88% cumulative average mortality in the groups C, V, CT, B, L, and T, respectively, and the mortality was random between pens, with no significant treatment effect.

There was no significant difference in the digestibility of dry matter (DM), ether extract (EE), and retention of crude protein (CP) among the groups (Supplementary Table 3).

### Microbiome Sequencing

The 16 S amplicon sequencing generated 82,35,312 raw reads with 44,46,248 quality-passed (after chimera removal and merging of quality-passed reads) consensus sequences or contigs (ranging from 74,090 to 3,73,945 per sample and an average of 2,47,013 per sample).

The shotgun sequencing generated about 1 billion raw reads corresponding to 157 GB of raw data from the gut content of the 24 chickens. After merging paired-end reads, filtering low-quality sequences, and host sequence removal, the average number of quality-controlled sequences per sample was 149,59,665 (range, 81,19,571-295,54,488).

### Operational Taxonomic Unit Occurrence

From the 16 S amplicon sequencing data, a total of 181,120 similarity-based operational taxonomic units (OTUs) were identified from 44,46,248 quality-passed chimera checked consensus reads. From 181,120 OTUs, 168,910 OTUs with less than 5 members were removed and 12,210 OTUs were selected for further analysis to focus on important OTUs only, to remove potentially spurious OTUs, and to improve the downstream statistical analysis.

From the shotgun sequencing data, a total of 39,986 phylotype OTUs were detected, out of which 33,891 phylotype OTUs were bacterial OTUs. The average number of bacterial taxonomic OTUs detected in different groups was comparable (ranging from 16,701 to 17,862; P > 0.05). In total, 18,163 OTUs contained ≤ 4 members or were prevalent in only 1 sample and 835 OTUs had ambiguous phylum-level classification. The average number of non-rare (having at least 5 members per OTU and with prevalence greater than 1 sample) bacterial taxonomic OTUs having valid phylum-level classification were also comparable between the groups (ranging from 13,918 to 14,730).

Based on Good’s coverage index, more than 99.9% of gut bacterial diversity was covered in all the groups.

### Taxonomy Assignment

**FIGURE 1:**
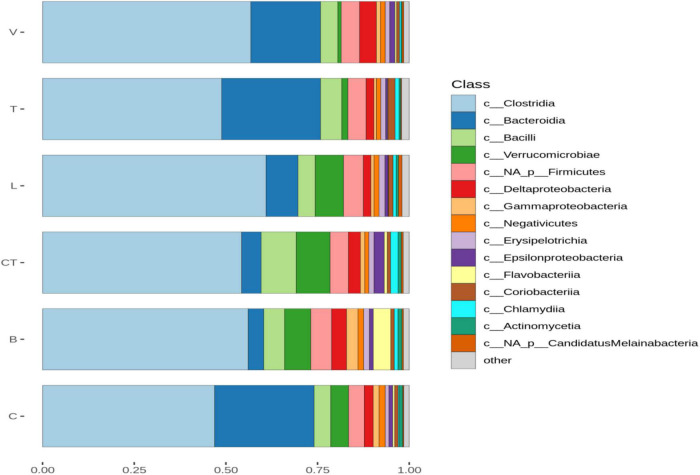
Barplots of the average normalized relative abundance of the 15 most abundant bacterial taxa identified to class level, found in different groups. “Not-assigned” are taxa identified to a lower taxonomic level than class, “Others” taxa not included in the 15 most abundant taxa.

Based on the shotgun sequence data, Clostridia were the most dominant class in all the groups (Figure 1). The average proportion of Bacteroidia was lower in the L, CT, and B groups as compared to the other groups including the C group. Whereas the proportion of Verrucomicrobia was higher in the L, CT, and B groups as compared to the other groups including C. The average abundance of unclassified Firmicutes and Flavobacteria was higher in the B group as compared to others. The average abundance of Epsilonproteobacteria and Chlamydia was higher in the CT group as compared to other groups. The average relative abundance of Bacilli was higher in the CT group as compared to others including the C group.

The taxonomic assignment of amplicon data (after removing OTUs with less than 5 members) resulted in the identification of 17 phyla, 157 genera, and 476 phylotype-OTUs (phylotype OTUs were obtained after merging distance-based OTUs with the same consensus taxonomy) being represented across the gut samples. In the C group, the gut microbiota was dominated by Lactobacillus (18.1%), Akkermansia (15.3%), Faecalibacterium (15%), Peptostreptococcaceae (13.5%), Lachnospiraceae (8.5%), Ruminococcaceae other than Faecalibacterium (7%) Methanobrevibacter (4.9%), *E. coli* (2.8%), and Desulfovibrio (2.3%) (Supplementary Presentation 1). In the BMD group, the proportion of Lachnospiraceae (22.8%), Faecalibacterium (18%), and Lactobacillus (15%) were higher with proportionately lesser populations of Akkermansia (2.4%), *E.coli* (<0.1%), Methanobrevibacter (<0.1%), and Desulfovibrio (<0.1%) as compared to the C group. In the CTC group, Peptostreptococcaceae (26.9%) and Faecalibacterium (20.7%) were higher with reduced populations of Akkermansia (1.7%), *E.coli* (<0.1%), Methanobrevibacter (<0.1%), and Desulfovibrio (<0.1%) as compared to the C group. In the LIN group, Allistipes (26.1%) were higher with a much lesser proportion of Lactobacillus (7.9%), Faecalibacterium (6.8%), *E. coli* (<0.1%), Methanobrevibacter (<0.1%), and Desulfovibrio (<0.1%) as compared to the C group. In the TYL group, the proportion of Faecalibacterium (38.5%) and Alistipes (22.1%) were higher with a much lesser population of Lactobacillus (4.5%), *E.coli* (<0.1%), Methanobrevibacter (<0.1%), and Desulfovibrio (<0.1%) as compared to C. In the case of VIR, the proportion of Lachnospiraceae (32.4%) and Lactobacillus (26.4%) increased with a reduction in the proportion of Faecalibacterium (3.2%), Peptostreptococcaceae (2.9%), and *E. coli* (< 0.1%) and a slight decrease in the proportion of sulfate reducing bacteria (2.2%) and Methanobrevibacter (1.2%) as compared to C.

**FIGURE 2:**
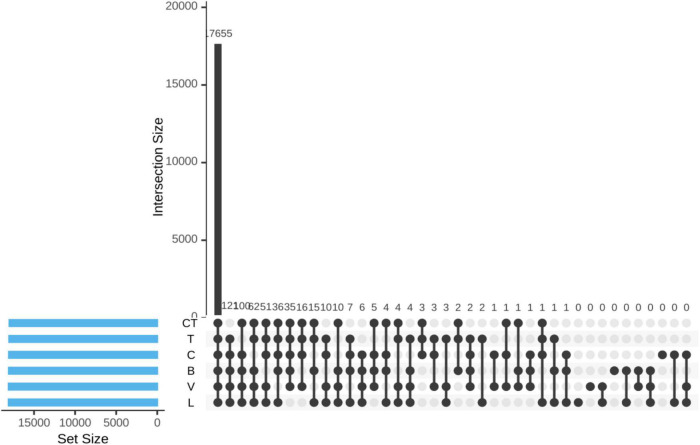
UpSet diagram visualizing intersections of sets of OTUs between different groups.

The UpSet diagram indicated a very high level of overlap of phylotype-OTUs among different groups (based on the shotgun sequence data) where most of the non-rare OTUs (17655 out of 18163) were detected in all the groups (Figure 2). A total of 121,100, 62, 51, 36, and 35 OTUs were not detected in the CT, T, C, B, V, and L groups, respectively, but were detected in all other remaining groups.

### Microbial Alpha Diversity and Data Rarefaction

Different alpha diversity metrics (the diversity within each group) were estimated to assess different aspects of the community structure.

The alpha diversity indices (ACE, Chao1, Fisher, Shannon, and Simpson) calculated on the amplicon sequencing data did not differ significantly among the groups. (Supplementary Table 4).

Based on the shotgun sequencing data, the alpha diversity (which takes into account both richness and evenness) estimator with the Shannon and Simpson index was higher (*P* < 0.01) in the CTC group as compared to the control (C) group. The Pielou’s evenness index was significantly higher in the CTC group and tended to be higher in the L group as compared to the C group (Supplementary Figure 1).

**FIGURE 3:**
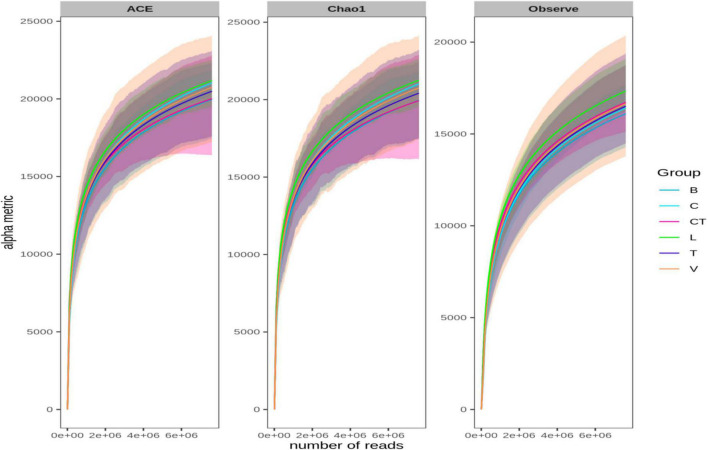
Rarefaction curves based on Chao1, ACE, and observed OTUs. Bacterial sequences were rarefied to the minimum library size (at 76,07,352 sequences per sample) without data filtering for rare OTUs.

Besides these estimators, rarefaction curves based on the observed species richness, Chao1, and ACE estimators were also plotted based on the shotgun data (Figure 3) and also on amplicon sequencing data (Supplementary Figure 2). The rarefaction curve depicts the correlation between the number of sequences and the number of taxonomic OTUs, and the steeper the slope, the higher the diversity (Heck et al., 1975). The rarefaction curve approached the asymptotic level for each group even on rarefying data to minimum library size, suggesting the availability of sufficient reads to represent each microbial community.

### Microbial Beta Diversity

The beta diversity (the partitioning of biological diversity among groups along a gradient, e.g., the number of species shared between two groups) analysis was undertaken to assess the relationship between microbial communities of different groups using different metrics to calculate the dissimilarity/distance matrix, such as Bray-Curtis, Jensen-Shannon, and Jaccard Index.

Beta diversity analysis using PCoA or NMDS plot on the amplicon sequencing data did not result in any visual separation of samples due to groups. PERMANOVA tests performed using all beta diversity metrics used in this study showed no significant (*P* > 0.05) differences in the community structure among different groups. At the phylotype-OTU level, Jensen Shannon-based PERMANOVA analysis had the highest Pseudo–F (1.1368) and R^2^ (0.321) values among all distance metrics indicating that only 32.1% of microbiota variation is explained by this category (group) besides a non-significant *p*-Value (*P* > 0.05). The beta dispersion values (PERMDISP) were non-significant for all groups in all diversity metrics analyzed at the phylotype-OTU level indicating homogeneous dispersion among groups.

**FIGURE 4:**
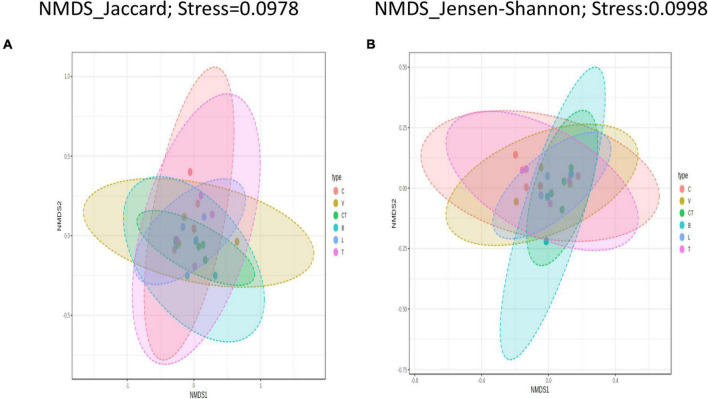
Beta diversity among treatments. Beta diversity plots visualized using Non-metric multidimensional scaling-based ordination at OTU level for different beta diversity metrics **(A)** Jaccard index, **(B)** Jensen-Shannon. A stress value of less than 0.1 represents a satisfactory-quality ordination. C, control; V, Virginiamycin; CT, Chlortetracycline; B, Bacitracin Methylene Disalicylate; L, Lincomycin; T, Tylosin.

Beta diversity analysis on the shotgun sequence data was visualized using nMDS as well as PCoA ordination methods, but due to space limitations, only plots obtained using nMDS are presented (Figure 4).

Permutational multivariate analysis of variance tests performed using all beta diversity metrics used in this study showed no significant (*P* > 0.05) differences in the community structure between different groups. At the phylotype-OTU level, the Jaccard Index based PERMANOVA analysis had the highest Pseudo–F (0.965) and R^2^ (0.211) values among all the distance metrics indicating that only 21.1% of microbiota variation is explained by this category (group) besides a non-significant *p*-value (*P* > 0.05). The beta dispersion values (PERMDISP) were non-significant for all groups in all diversity metrics analyzed at the phylotype-OTU level indicating homogeneous dispersion among groups.

The NMDS scaling based on all three distance metrics showed a clear visual separation of groups at the phylotype-OTU level, but there was a high degree of overlap between the groups.

### Differential Abundances of Bacteria

DESeq2 analysis of CSS normalized amplicon sequencing data (OTUs with at least 5 members) with FDR correction indicated that 5 phyla, 7 classes, 5 families, 6 genera, and 20 OTUs were significantly different in abundance between groups. At the class level, Deltaproteobacteria, Methanobacteria, Gammaproteobacteria, Actinobacteria, Mollicutes, Campylobacteria, and Bacteroidia differed significantly in abundance between groups. The abundance of genera, Desulfovibrio, Clostridium_sensu_stricto1, and Methanobrevibacter was significantly lower in all AGP groups except virginiamycin as compared to the C. The abundance of the genus level uncultured group Ruminococcaceae_UCG_010 was significantly higher in the Tylosin group as compared to C. The abundance of the genus, Escherichia was significantly lower in all the AGP groups as compared to C. The abundance of the genus, Pontibacter was significantly lower in the groups, tylosin, and lincomycin as compared to C.

**FIGURE 5:**
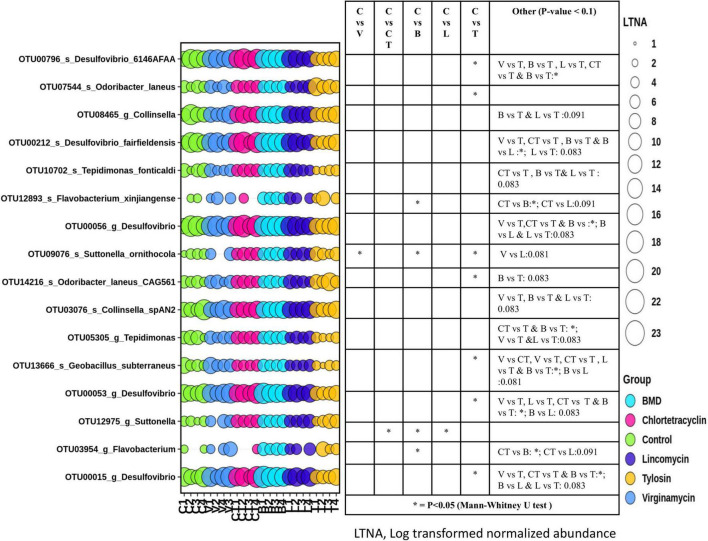
Differential abundance of gut microbiota in different groups at OTU level. OTUs with significant difference in abundance among groups identified with DESeq2 and passing false discovery rate (FDR) filter, were plotted. The size of the bubbles in the bubble plot indicates the log- transformed (LN(2)) normalized (cumulative sum scaling) abundance of each OTU.

DESeq2 analysis of CSS normalized shotgun sequencing data (18,163 OTUs with at least 5 members) with FDR correction indicated that only 15 phylotype-OTUs were significantly different in abundance among the groups (Figure 5). Comparisons between pairs of groups using the Mann–Whitney U test indicated that there was a significant difference in the abundance of many phylotype-OTUs between different groups. Interestingly, the abundance of phylotype-OTUs, such as OTU00796 (species *Desulfovibrio* 6146AFAA) and OTU13666 (species *Geobacillus subterraneus*) was lower in the T group as compared to the C group, but the abundance of OTU07544 (species, *Odoribacter laneus*) was higher in the T group than in the C group. The abundance of the OTU12975 (genus *Suttonella*) was higher in the CT group as compared to the C group. The abundance of OTU12893 (species *Flavobacterium xinjiangense*) and OTU09076 (species *Suttonella ornithocola*) were higher in the L group as compared to the C group. The abundance of OTU09076(species *Suttonella ornithocola*) was also higher in the V group as compared to the C group.

### Differential Abundance of Bacteriophages

**FIGURE 6:**
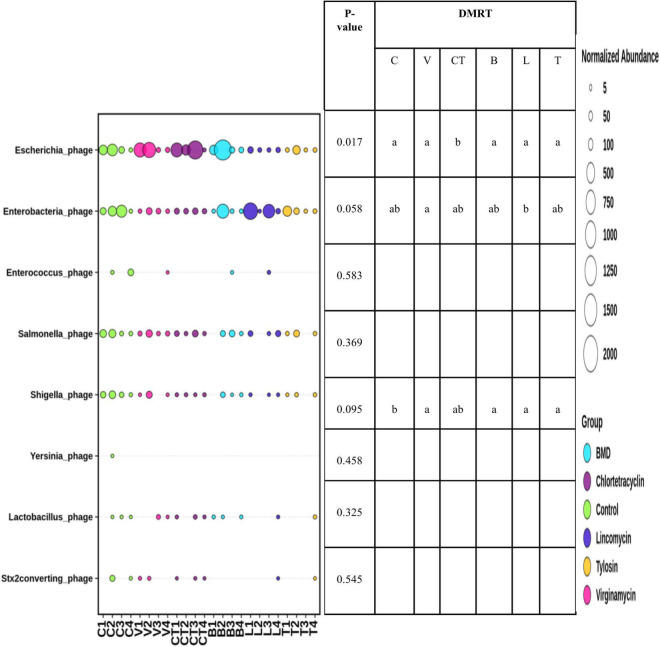
Differential abundance of gut bacteriophages in different groups. The size of the bubbles in the bubble plot indicates the normalized (normalized to per million reads) abundance.

The abundance of Escherichia phage increased significantly in the group supplemented with CTC as compared to C and other antimicrobials (Figure 6). The abundance of other phages was not influenced significantly by the supplementation of AGP in the diet. However, the abundance of Enterobacteria phage tended (*P* < 0.1) to be influenced by AGP supplementation with the highest abundance in the LIN group. On the other hand, the abundance of Shigella phage tended to be lower in most of the AGP groups.

### Differential Abundance of Antimicrobial Resistance Genes

**FIGURE 7:**
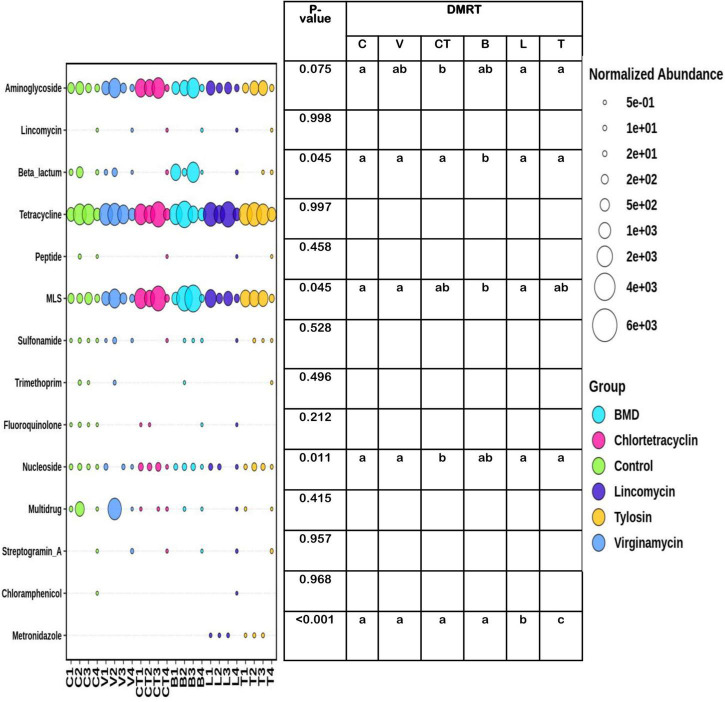
Differential abundance of different categories of gut antimicrobial resistance genes in different experimental groups. The size of the bubbles in the bubble plot indicates the normalized (normalized to per million reads) abundance. MLS, macrolide-lincosamide-streptogramin B.

In total, 62 ARGs from 15 ARG classes were detected (Supplementary Table 5). The abundance of tetracycline genes was high across the groups and was not influenced by the inclusion of AGPs in the diet (Figure 7). In general, the abundance and prevalence of aminoglycoside, tetracycline, and macrolide-lincosamide-streptogramin B (MLS) resistance genes were higher than other classes of AMR genes. The abundance of aminoglycoside resistance genes tended to be higher in the group supplemented with CT as compared to C, L, and T groups. The abundance of nucleoside resistance genes was significantly higher in the group supplemented with CT as compared to C, L, and T groups, whereas in group B it was at an intermediate level. The proportion of beta-lactam and MLS resistance genes increased significantly after supplementing B in the diet as an AGP. The abundance of metronidazole resistance genes increased significantly in the group supplemented with L and T as an AGP. The abundance of resistance genes associated with the resistance of lincomycin, peptide antibiotics, sulfonamide, trimethoprim, fluoroquinolone, chloramphenicol, and multidrug resistance categories were not influenced significantly by the use of the AGPs in the diet and in general, had lower prevalence and abundance levels across the groups.

### PCR- and Quantitative Real-Time PCR-Based Differential Abundance of Antimicrobial Resistance Genes

On conventional PCR analysis, bla-CTX-M genes were not detected in samples (6 samples/group) except for those from the third feeding cycle in the C group (Supplementary Table 6). Similarly, colistin and the MDR (efflux pump) genes were not detected in any of the samples. However, ampicillin (beta-lactamase) resistance was detected in most of the samples, and in the case of V, CT, and B groups, the occurrence of *bla*TEM resistance in the third feeding cycle was higher than in the first cycle. Quinolone resistance gene *qnr*A detected in some of the samples in the first cycle was not detected in the third cycle in the case of T, L, and CT but was detected both in the first and third cycles in the C group. Another quinolone resistance gene, qnrB gene was detected in most of the groups in the third cycle and not in the first cycle. Quinolone resistance gene, gepA was detected in moderate to a high level of prevalence in both the cycles in case of C, V, CTC, and L samples but was not detected in B and T groups.

The qPCR analysis of samples found to be positive in conventional PCR indicated that for *bla*_*TEM*_ resistance, copy number in third cycle was higher than in the first cycle for most of the groups, but for quinolone resistance gene, *qnr* B the reverse was true (Supplementary Table 6).

### Effects on the Abundance of *Escherichia coli* and Lactobacillus in Gut Content

Based on qPCr assay, during the first cycle, the CT group had a significantly lower density of *E. coli* as compared to C (Table 2). The other three AGPs namely V, B, and L tended to have lower *E. coli* density, whereas the T group had comparable levels of *E. coli* with that of the C group. During the third cycle, V and L had a significantly lower density of *E.coli* as compared to C, and T and B groups had intermediate levels whereas the CT group had a comparable level of *E. coli* with that of the C group. The density of Lactobacillus spp. was not significantly influenced by the supplementation of AGP.

**TABLE 2:** Effects of treatments on the abundance of *Escherichia coli* and Lactobacillus *spp. in gut content.*

Treatment	*E.coli* (Log_10_ no of copies of rrs gene/50 ng DNA)		*Lactobacillus spp.* (Log_10_ no of copies of rrs gene/50 ng DNA)	
	Cycle 1	Cycle 3	Cycle 1	Cycle 3
C	3.738*^bc^*	4.485*^c^*	4.361	3.342
V	3.104*^ab^*	2.838*^a^*	4.547	4.423
CT	2.703*^a^*	4.618*^c^*	4.818	3.553
B	3.156*^ab^*	4.322*^bc^*	4.493	3.878
L	3.411*^ab^*	2.659*^a^*	4.662	2.825
T	4.355*^c^*	3.428*^ab^*	4.889	3.462
SEM	0.249	0.321	0.135	0.158
N	6	6	6	6
*P*-value	0.001	<0.001	0.891	0.070

*V, Virginiamycin; CT, chlortetracycline; B, bacitracin methylene disalicylate; L, lincomycin; T, tylosin; N, number of replicate pens; means bearing common superscript does not differ significantly (P < 0.05).*

## Discussion

In the present study, supplementation of broiler diets with AGPs showed no growth-promoting effect except for the 1–21 days period in one out of the three trials. Supplementation with AGPs also did not influence the apparent digestibility of nutrients.

Based on statistical power curve analysis, Pesti and Shim (2012) have shown that for a broiler chicken experiment, testing for a difference in BW for 2 groups having mean BW of 1,731 g and SEM of 19 g and mean FCR of 1.865 g feed/g gain with an SEM of 0.015 g/g with 12 replicate pens per group would be expected to conclude a significant difference of 80% of the time if the differences were 4.6% in BW and 3.4% in FCR. Using the means and SEMs from our current study and following the method of Pesti and Shim (2012), it could be expected that 12, 13, and 14 replicates with 5 birds per pen can detect about 71, 68, and 65 g difference in BW between two groups at 5% level of significance at 80% of the time, and hence our experiments had adequate power to detect a significant difference that can be considered important commercially. In two out of the three trials/cycles, the BWG of the negative C group was higher than any of the AGP groups. In the remaining cycles, the difference in BWG between the best performing AGP group and the negative C at the 35th day is only 39 g. Hence, a higher number of replicates in feeding trials would unlikely change the inference or indicate a significantly better performance due to the inclusion of AGP. Hence, the main reason for not getting a significant difference is the less difference between treatment means obtained rather than the lack of statistical power due to replicates.

Considerable variability in response to AGP use has been reported. Many studies have shown no weight gain difference in broilers fed with an AGP diet in the absence of health problems (Denev, 2006; El-Faham et al., 2015; Naveenkumar et al., 2017). However, other studies have reported positive effects of AGP on broiler weight gain or feed conversion (Zhang et al., 2005; Peng et al., 2016; Salaheen et al., 2017; Wu et al., 2018). In a meta-analysis involving 174 scientific articles containing 183 experiments on broiler chicken, Maria et al. (2019) reported that higher weight gain and lower feed conversion ratio were observed in AGP fed groups during the initial phage (1 to 21 days), and the total period (1 to 42 days) with no difference in the final phase (22 to 42 days). A reduction in the effectiveness of AGPs in the last 30 years was suggested by Laxminarayan et al. (2015), which may be linked to the optimization of production conditions, better nutrition, increase in the baseline weight gain of animals, and increasing levels of antimicrobial resistance.

Antimicrobial growth promoters are postulated to affect performance through the modulation of the intestinal microbiome. The microbial communities differ in the gastrointestinal tract of the chicken (Sekelja et al., 2012). Here, we analyzed microbiota from the entire hindgut (duodenum to cloaca including cecum) to focus on segments generally considered to be important for gut health and function. Samples from the entire hindgut were mixed and, considering that we had modest sequencing depth, it can be safely assumed that microbes from all hindgut segments are adequately represented. Further, as we have mixed the gut content similarly for all of the groups, the mixing is unlikely to impact results with respect to the ability to compare community composition and diversity among the groups.

The advent of high throughput sequencing and molecular tools has allowed for a detailed characterization of the gut microbiota of chicken quickly and robustly, without the need to culture the microorganisms. Traditionally, sequence reads are clustered into OTUs at a defined identity threshold to avoid sequencing errors generating spurious taxonomic units. However, recently amplicon sequence variants (ASVs) have been proposed as an alternative to OTUs wherein a denoising algorithm is employed to generate an error model based on the quality of sequencing run to distinguish between predicted true biological variation and that is likely to be generated by a sequencing error. Several studies have compared the result of OTU-based and ASV-based approaches. Studies involving mock communities indicated that ASV-based approaches had a higher sensitivity than OTU-based approaches in detecting the rare bacterial strains present, sometimes at the expense of specificity (Prodan et al., 2020). However, studies utilizing real biospecimens from different ecosystems including chicken gut indicated that OTU-based and ASV-based approaches resolved similar biological signals, beta diversity, community composition, and taxonomy profiles (Allali et al., 2017; Glassman and Martiny, 2018; Joos et al., 2020; Moossavi et al., 2020). Further, recently it has been shown that the ASV approach can lead to conflicting inferences about the ecology of some bacterial genomes due to the presence of multiple copies of the 16S rRNA gene with a slightly varying sequence within the genome of a particular species of bacteria as ASV is sensitive to even single nucleotide difference (Schloss, 2021). In this study, our aim was not to detect rare organisms but to focus on the most important ones with adequate abundance. Most of the rare organisms would be removed in typical data filtering steps before differential abundance analysis and practically may not contribute substantially to the overall gut health or function. Hence, we have utilized the traditional OTU-based approach rather than the ASV-based approach for clustering sequence reads in our analysis.

The chicken gut harbors a range of microorganisms that have been proven to have a strong impact on the performance and health of the chicken. Adverse, pathogenic, and potentially pathogenic bacteria in the gut of chicken, such as *E. coli*, Salmonella spp., and *C. perfringens*, compete with the host for nutrients and may also damage the intestinal epithelium, which adversely affects the digestion and absorption function of the host and invade the host system causing morbidity, performance loss, and mortality (Gunal et al., 2006), besides posing a risk of spreading zoonotic diseases to humans. Although many *E. coli* strains are commensals, an unambiguous distinction between pathogenic and commensal strains is not so easy and may require a thorough analysis of a large number of virulence genes (or genome) and phenotype traits, as MiSeq data on 16S rRNA gene cannot differentiate them. Further, it has been emphasized that the thin borderline between commensalism and pathogenicity can be blurred by gene transfer, mutation, etc., due to the high plasticity of the genome of *E. coli* (Leimbach et al., 2013).

On the other hand, there are certain beneficial bacteria in the gut that provide health benefits, including enhancing the function of the intestinal barrier of the host, excluding potential pathogens, and maintaining homeostasis in the gut. Such bacteria are also called probiotic bacteria. Certain probiotic gut bacteria can also produce bacteriocins (a group of antimicrobial peptides) to selectively inhibit the growth of other bacteria including pathogenic bacteria. Various strains of Lactobacillus spp. isolated from the chicken gut were shown to produce bacteriocins, which are inhibitory to various bacteria, including pathogens, such as Campylobacter, *Salmonella enteritidis*, and *C. Jejuni* (Svetoch et al., 2011). The lactic acid produced by Lactobacillus can be converted to beneficial short-chain fatty acids (SCFAs), such as acetate, propionate, and butyrate by certain gut bacteria which in turn supply energy to the host and gut epithelial cells. It has been shown that there is a high correlation between the abundance of beneficial microbes, such as Lactobacillus in the gut with feed efficiency in chickens (Yan et al., 2017). It was reported that Lachnospiraceae and Ruminococcaceae are associated with good gut health through SCFA production and fiber degradation and hence have probiotic properties and are categorized as beneficial bacteria (Biddle et al., 2013; Stanley et al., 2016). Similarly, emerging evidence indicates that bacteria belonging to Faecalibacterium spp. have an important role in maintaining SCFA production, gut immunity, and healthy metabolism and are considered probiotic and beneficial bacteria (Miquel et al., 2013).

Earlier, based on the amplicon sequencing of gut content, we observed that *E. coli* was the most abundant bacteria among the known potential pathogenic bacteria and Lactobacillus was one of the most abundant beneficial bacteria with a known probiotic role in broiler chicken (Paul et al., 2021a). Hence, we have utilized *E. coli* and Lactobacillus as representatives of potentially pathogenic and beneficial bacteria, respectively, for qPCR assay.

The taxonomic assignment of amplicon sequencing data indicated that individual AGPs had some significant effect on the gut microbiome. The BMD-enriched Lachnospiraceae without affecting beneficial bacteria with known probiotic roles, such as Faecalibacterium and Lactobacillus. CTC enriched Peptostreptococcaceae and Faecalibacterium and depleted Akkermansia. Tylosin enriched Faecalibacterium and Allistipes, while reducing the abundance of Lactobacillus. Lincomycin enriched Allistipes and depleted both Lactobacillus and Faecalibacterium. Virginiamycin enriched Lachnospiraceae and Lactobacillus while depleting Akkermansia and Faecalibacterium. All the AGPs except Virginiamycin reduced the relative abundance of sulfate-reducing bacteria and methanogens. However, differential abundance analysis using DESeq2 and strict statistical screening methods like FDR adjustment has shown significant differences between groups in a limited number of OTUs and in a few genera. *E. coli* was the most abundant, potentially pathogenic bacteria in the gut of broiler chickens in the C group. There was a significant decrease in the abundance of potentially pathogenic genera like Escherichia in all the AGP groups as compared to C. Our qPCR data have also indicated that most of the AGPs had significantly lower or a trend of lower *E. coli* density during the first cycle as compared to the C. This is in agreement with the study of Unno et al. (2015) who also observed that although no growth performance enhancements were observed in pigs with AGPs, such as chlortetracycline, sulfathiazole, and penicillin, the use of AGPs inhibited the potential pathogens in the gut of swine.

*Escherichia coli* has been found as a part of normal flora in the gastrointestinal tracts of chickens and is considered one of the most important and frequent pathogens responsible for food-borne diseases in poultry and humans worldwide (Akbar et al., 2014). Hence, a decrease in *E. coli* in the gut of the chicken with the use of AGP may be important for poultry producers. However, a reduction in *E.coli* alone may not justify the use of AGPs in poultry production.

Based on the shotgun sequencing data, Clostridia under the phylum, Firmicutes was the most dominant class in all the groups. There was a considerable variation between individual birds in the relative abundance of different classes. Statistical analysis revealed no significant difference in the relative abundance of Clostridia or Firmicutes among treatments. However, the average proportion of Bacteroidia was lower in some of the AGP groups. The Upset diagram indicated that most of the non-rare OTUs were detected in all the groups and only a few OTUs were not detected in each of the AGP groups indicating that the AGPs influenced only a few OTUs. In the present study, richness indices were not affected by supplementation of AGP, but the evenness and diversity were higher in the chlortetracycline group than that in the C group. Early culture-based and culture-independent studies using molecular tools, such as denaturing gradient gel electrophoresis and Sanger sequencing of clone libraries revealed AGP- induced shifts in microbiota composition (Dumonceaux et al., 2006; Lin et al., 2013). Subsequent next-generation 16 S amplicon sequencing or shotgun metagenomic sequencing also demonstrated specific changes in certain bacterial communities but in many studies, results were inconsistent. Several studies have indicated no effect of AGPs on alpha diversity (Danzeisen et al., 2011; Proctor and Phillips, 2019), while others have indicated a decrease (Choi et al., 2018; Diaz Carrasco et al., 2018) or increase (Crisol-Martinez et al., 2017) in alpha diversity metrics. Robinson et al. (2019) reported a significant decrease in richness and a concurrent increase in evenness in cecal microbiota on 2-week supplementation with monensin and salinomycin. In the present study, supplementation of the AGPs had no significant effect on beta diversity. In contrast, beta diversity metrics were shown to be consistently influenced by AGPs across many studies (Costa et al., 2017; Crisol-Martinez et al., 2017; Diaz Carrasco et al., 2018) with only a few studies reporting no shift (Pourabedin et al., 2015).

Based on shotgun sequences, the abundance of only 16 OTUs differed significantly between at least one pair of the groups. The most notable effect of AGP supplementation was the differential enrichment of Odoribacter (in case of T), Flavobacterium (in case of B), and Suttonella (in case of V, B, CT, and L) and depletion of Desulfovibrio (in case of T) and Geobacillus (in case of T) as compared to C. Bacteria within the genus Odoribacter, belonging to the order Bacteroidales, are SCFA-producing members of gut microbiota. Recent *in vitro* studies have indicated that some members of this genus could potentially exert anti-inflammatory action in the gut epithelium and are likely to be commensal with primary beneficial interactions with the host (Hippala et al., 2020). Flavobacteria, belonging to the phylum Bacteroidetes, have been reported from many different ecological niches including the gastrointestinal tract of animals and they are well-known degraders of polymeric organic matters and gut bacteroidetes have been shown to interact with the immune system for the activation of T-cell mediated responses (Wen et al., 2008) and limiting the colonization of GIT by potentially pathogenic bacteria (Mazmanian, 2008), and are known to produce butyrate which plays a role in maintaining healthy gut (Thomas et al., 2011). Geobacillus, belonging to the class Bacilli, are known to produce a wide range of secretary compounds including nitric oxide having biological effects on GIT (Ilinskaya et al., 2017). *Suttonella ornithocola*, first isolated from the lungs of the British tit species belonging to the family, Cardiobacteriaceae is believed to be a primary pathogen in some of the wild birds (Lawson et al., 2011), and its role in chicken gut is not known. Its enrichment in the case of V, B, and L supplemented groups as compared to C can be considered an adverse effect of AGP supplementation.

Desulfovibrio spp. are sulfate reducers and are potential hydrogen sinks which not only facilitate anaerobic fermentation but also produce hydrogen sulfide which is considered toxic (Murros et al., 2021). Thus, depletion of Desulfovibrio spp. in case of supplementation with T is expected to exert a mixed effect on gut health and fermentation.

The present study indicated that the abundance of Escherichia phages increased significantly with supplementation of CTC and that other AGPs did not influence bacteriophages significantly. Reports on the effects of AGPs on the abundance of bacteriophages in the gut are limited. Salaheen et al. (2017) observed that AGP supplementation (tylosin, neomycin, bacitracin, erythromycin, and oxytetracycline) was associated with a higher relative abundance of bacteriophages.

In the present study, the tetracycline resistance genes were the most abundant followed by MLS and aminoglycoside resistance genes and this is the first metagenome-based analysis of the prevalence of ARGs in Indian broilers. However, a recent report on antibiotic susceptibility analysis showed that 77 and 100% of bacterial isolates belonging to the *E. coli* and *Klebsiella pneumonia* isolated from the fecal samples of chicken from 5 different Indian farms were resistant to tetracycline, and further molecular screening for *tetA* and *tetB* genes showed 85% of isolates to have tetA and 22% isolates with tetB (Sreejith et al., 2020). The tetracycline resistance was also the most prevalent in the fecal samples collected from cattle (Zaheer et al., 2018) and humans (Forslund et al., 2013; Hu et al., 2013) in other counties. In the present study, many antibiotic resistance genes were ubiquitous in the gut content of the chicken regardless of antibiotic administration. However, different AGPs increased the abundance of different ARGs, some of which are structurally unrelated to the AGPs being fed to chicken. For example, bacitracin (peptide antibiotic) significantly increased the abundance of beta-lactam resistance genes. Similarly, the groups fed with bacitracin (peptide antibiotic) and chlortetracycline (tetracycline group) had a high abundance of MLS class ARGs, such as *erm* (erythromycin resistance), *lnuB* (confers resistance to lincomycin and clindamycin) and *msrB* (confers resistance to erythromycin and streptogramin B or quinupristin) and nucleoside class of ARGs, such as *sat4* and *sat2*. Earlier, the increasing use of virginiamycin in animals as a feed additive has been shown to be associated with higher rates of resistance to virginiamycin, and quinuprustin–dalfopristin (streptogramin compounds) has been associated with increased abundance of *vat*, and *vatG* genes (Soltani et al., 2000). Quinupristin-dalfopristin is a semi-synthetic mixture of streptogramin A and B, which has been used in the treatment of infections caused by multidrug-resistant pathogens including vancomycin-resistant *Enterococcus faecium*. In this study, *vatE* genes were detected in limited numbers and also in lower prevalence. Moreover, the density of streptogramin resistance genes in the virginiamycin group was not significantly different from that of the C group. Similarly, lincomycin feed did not influence the abundance of lincosamide resistance genes or any other class of antimicrobials. However, lincomycin significantly increased the abundance of metronidazole resistance genes. Tylosin (macrolide group antibiotic) was associated with an increased abundance of resistance to the structurally related antibiotic erythromycin and also that of metronidazole, which is not structurally related to it. Thus, the present study indicates that feeding of AGPs modulates the selection of ARGs in a complex manner and may not be explained by simplistic cause and effect relationships or structural similarity and may be due to genetic linkage. The use of tylosin is very common in poultry in India as besides its use as AGP, it is also used to treat or prevent the occurrence of chronic respiratory disease caused by *Mycoplasma gallisepticum*. Metronidazole resistance is not very common. Metronidazole has been a mainstay both for prophylaxis and treatment of anaerobic infections. Metronidazole resistance has been emerging worldwide, although presently its prevalence is about 5%. Such resistance has become a great concern because of limited therapeutic options available for treating anaerobes, such as Bacteroides infection (Sethi et al., 2019). Earlier, Sethi et al. (2019) reported that 31% of the human Bacteroides spp. isolates from North India were resistant to metronidazole and 53% of isolates were positive for *nim* gene. It may be mentioned that no genes related to colistin resistance were detected in any of the samples in this study in both qPCR and metagenomic shotgun analysis. PCR and qPCR analysis confirmed the presence of some of the ARGs identified through shotgun analysis. Earlier, Gupta et al. (2021) observed that although antibiotic resistance genes were ubiquitous in chicken cloaca and litter, bacitracin fed groups had higher levels of bacitracin resistance genes, and vancomycin-resistant bacteria and enrofloxacin treatments generally facilitated increased abundance of multidrug-resistant bacteria. In contrast, Danzeisen et al. (2011) did not observe any significant difference in antimicrobial resistance gene counts in the cecal content of chicken on supplementing with monensin, tylosin, and virginiamycin. Salaheen et al. (2017) observed that AGP supplementation (tylosin, neomycin, bacitracin, erythromycin, and oxytetracycline) was associated with unique cecal resistome compared with C. Earlier, Apata (2009); Diarra and Malouin (2014) reported that the use of antimicrobials as AGP, administered at a subtherapeutic dose usually over a longer period, may lead to the development of antimicrobial resistance. The abundance of ARGs detected in this study could be directly related to the changes in the gut microbiome but from metagenomic data, it is not easy to establish which bacteria are associated with which ARG.

## Conclusion

Our results demonstrate the high complexity of poultry gut microbiome and resistomes and the ubiquitous presence of different classes of ARGs in the gut of broiler chicken. Feeding of AGPs affects gut bacteria, pathogenic bacteria, and bacteriophage only to a limited extent. Feeding AGPs affects the abundance of ARGs in a highly complex manner which may not be explained by simple cause and effect relationships or structural similarities. More studies like this are required to determine baseline resistome levels in broiler production facilities and evaluate the effects of continuous use of different AGP classes. Nevertheless, the present study has indicated that the AGPs favor the selection of different ARGs even though that may not be structurally related to the AGP and feeding of AGPs had no significant effect on the performance of broiler chickens. The study highlighted the need to monitor the spread of ARGs and the setting up an action plan to reduce the use of AGPs in broiler production.

## Data Availability Statement

The datasets presented in this study can be found in online repositories. The names of the repository/repositories and accession number(s) can be found below: https://www.ncbi.nlm.nih.gov/bioproject/?term=PRJNA730210; https://www.ncbi.nlm.nih.gov/bioproject/?term=PRJNA817574.

## Ethics Statement

This study was carried out using welfare standards consistent with those established under Indian Law. All protocols were approved by the Institute Animal Ethics Committee of ICAR Directorate of Poultry Research (approval order no: IAEC/DPR/18/10 dated 29.9.2018).

## Author Contributions

SP and SR designed the study and executed the experiments. SP did data analysis, bioinformatic analysis, visualization, and manuscript writing. NH, SM, and MG carried out PCR analysis for ARGs. NW optimized PCR and qPCR protocols for ARG detection and did the manuscript revision. GR, VK, and PP recorded feeding trial data and analyzed feed and blood samples. RC and MR did the manuscript correction. All authors contributed to the article and approved the submitted version.

## Conflict of Interest

The authors declare that the research was conducted in the absence of any commercial or financial relationships that could be construed as a potential conflict of interest.

## Publisher’s Note

All claims expressed in this article are solely those of the authors and do not necessarily represent those of their affiliated organizations, or those of the publisher, the editors and the reviewers. Any product that may be evaluated in this article, or claim that may be made by its manufacturer, is not guaranteed or endorsed by the publisher.

## Footnotes

1
https://github.com/gisleDK/Biopythonpieces/blob/master/Scripts/kaiju2phyloseq.py

2
https://www.arb-silva.de/

3
https://github.com/YuLab-SMU/MicrobiotaProcess/

## Supplementary Material

Supplementary Figure 1 Violin plots representing the distribution of alpha diversity indices of bacterial sequences (cycle 3) without data filtering for rare operational taxonomic units (OTUs). The box plot shows the median, 25th, and 75th percentiles, and whiskers indicate 1.5 x interquartile range. *P*-values denote the level of significance on pairwise comparison using a non-parametric Wilcoxon test.

Supplementary Figure 2 Rarefaction curve based on the observed richness after the removal of OTUs appearing in only one sample and after rarefying data to the minimum library size.

Supplementary Table 1 Ingredient and nutrient composition of basal diets (common for all three trials).

Supplementary Table 2 Meat processing parameters during cycle 3 in different groups.

Supplementary Table 3 Effect of supplementing antimicrobial growth promoters on apparent total tract digestibility (nutrient retention) during experimental cycle 1.

Supplementary Table 4 Statistical analysis of alpha diversity measures for comparing different groups.

Supplementary Table 5 List of antimicrobial resistance genes detected in shotgun sequencing data.

Supplementary Table 6 Effects of different antimicrobial growth promoters (AGPs) on the prevalence of antimicrobial resistance genes (ARGs) as determined by conventional PCR and qPCR.

Supplementary Data Sheet 1 Details of PCR and qPCR protocols used for the detection of antimicrobial resistance genes in metagenomic DNA isolated from gut content.

Supplementary Presentation 1 Barplots of the average normalized relative abundance of the 25 most abundant bacterial taxa at different taxonomic levels, found in different groups. “Others” are taxa not included in the 25 most abundant taxa.
