# Supplementary material for: Effects of Dietary Antimicrobial Growth Promoters on Performance Parameters and Abundance and Diversity of Broiler Chicken Gut Microbiome and Selection of Antibiotic Resistance Genes
Source: Front Microbiol. 2022 Jun 16;13:905050. doi: 10.3389/fmicb.2022.905050 (PMC9244563; doi:10.3389/fmicb.2022.905050)
Supplement: Supplementary Figure 1 — Violin plots representing the distribution of alpha diversity indices of bacterial sequences (cycle 3) without data filtering for rare operational taxonomic units (OTUs). The box plot shows the median, 25th, and 75th percentiles, and whiskers indicate 1.5 x interquartile range. P-values denote the level of significance on pairwise comparison using a non-parametric Wilcoxon test. [file Image_1.pdf]

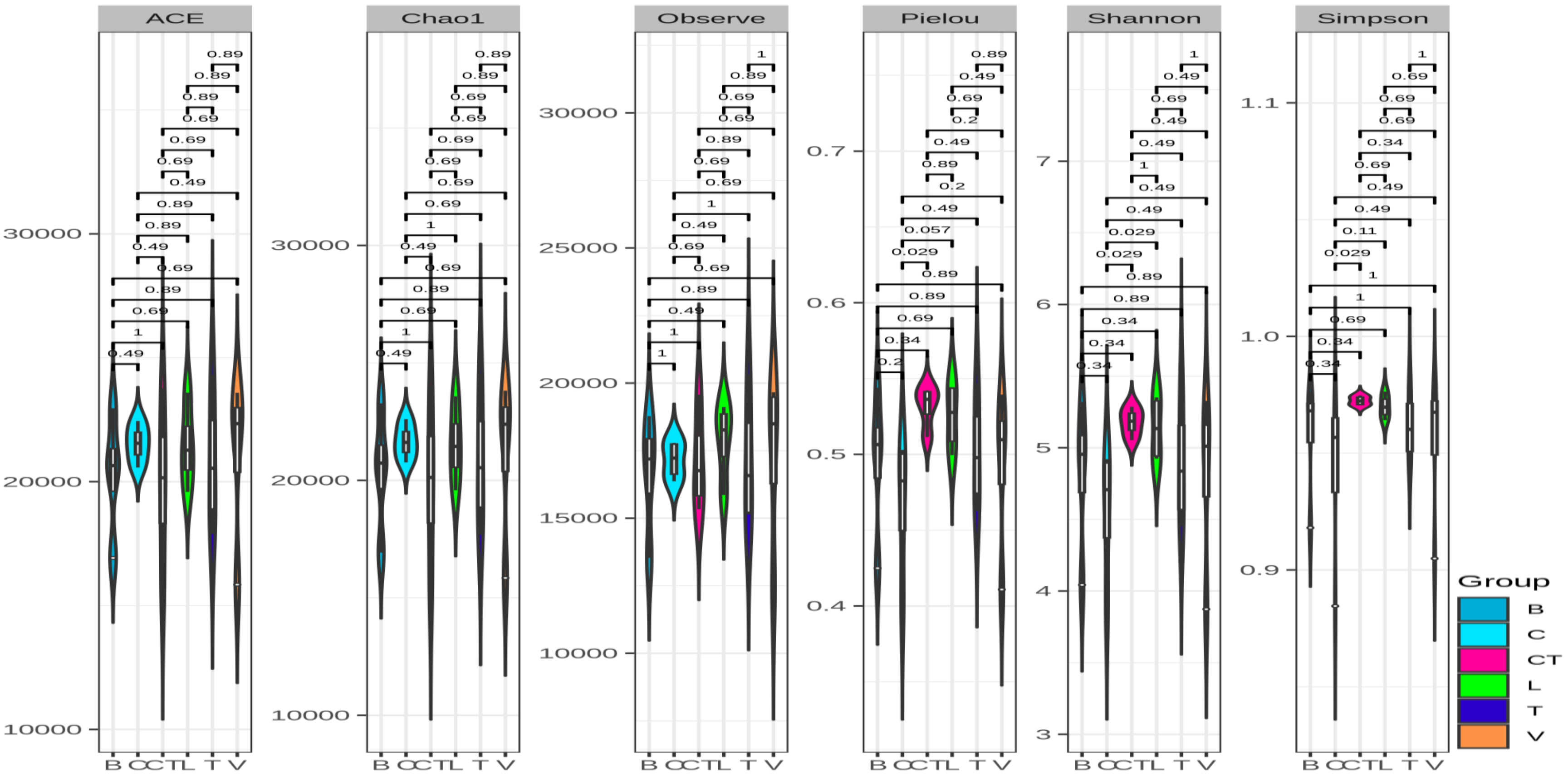

Supplementary Figure 1. Violin plots representing the distribution of alpha diversity Indices of bacterial sequences (cycle 3) without data filtering for rare OTUs . The box plot shows the median, 25th and 75th percentiles, and whiskers indicate 1.5 x interquartile range. P-values denote level of significance on pairwise comparison using nonparametric Wilcoxon test.
