# Supplementary material for: Effects of Dietary Antimicrobial Growth Promoters on Performance Parameters and Abundance and Diversity of Broiler Chicken Gut Microbiome and Selection of Antibiotic Resistance Genes
Source: Front Microbiol. 2022 Jun 16;13:905050. doi: 10.3389/fmicb.2022.905050 (PMC9244563; doi:10.3389/fmicb.2022.905050)
Supplement: Supplementary Figure 2 — Rarefaction curve based on the observed richness after the removal of OTUs appearing in only one sample and after rarefying data to the minimum library size. [file Image_2.pdf]

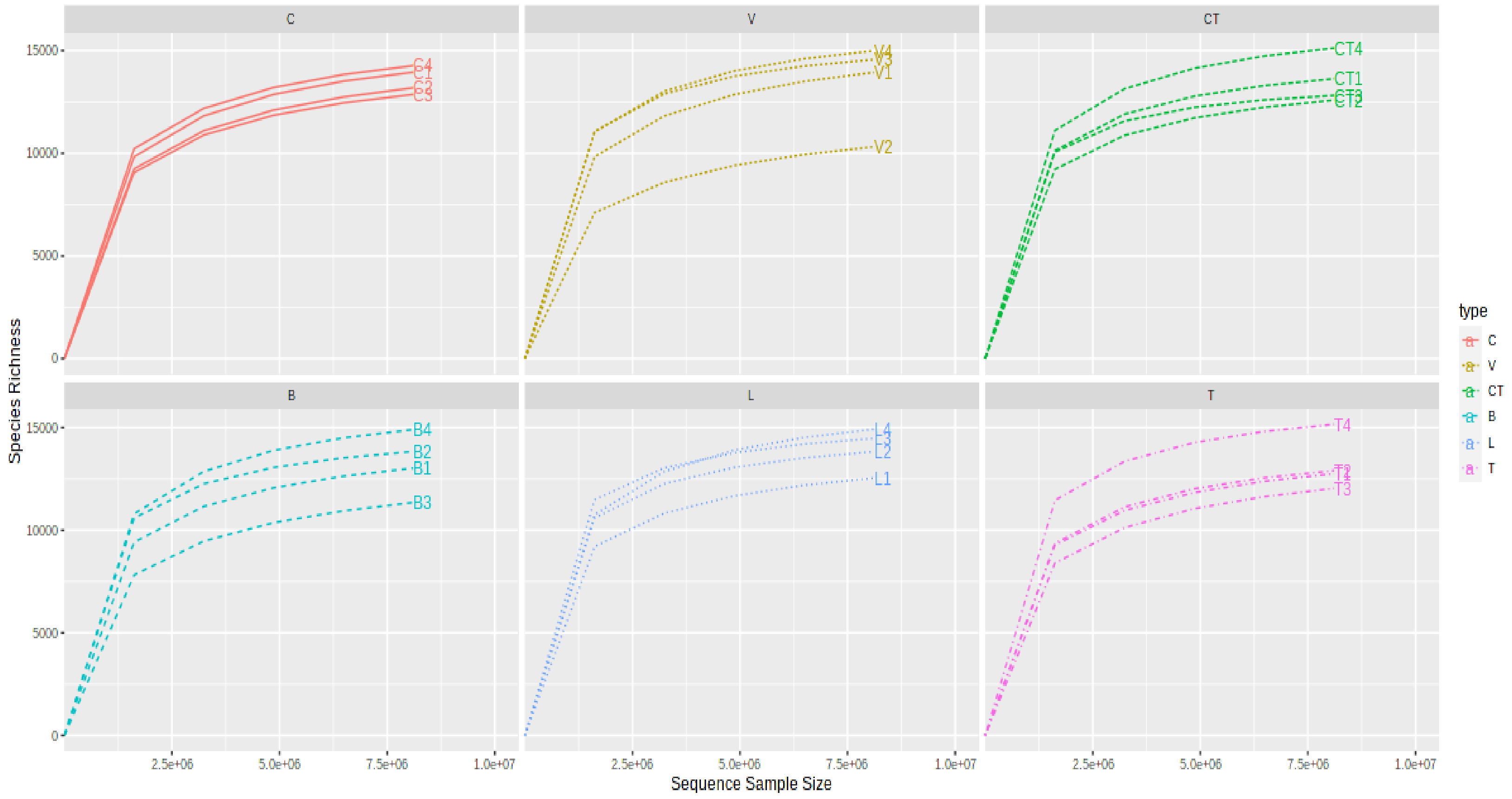

Supplementary Figure 2. Rarefaction curve based on observed richness after removal of OTUs appearing in only one sample and after rarefying data to the minimum library size
