# Supplementary material for: Effects of Dietary Antimicrobial Growth Promoters on Performance Parameters and Abundance and Diversity of Broiler Chicken Gut Microbiome and Selection of Antibiotic Resistance Genes
Source: Front Microbiol. 2022 Jun 16;13:905050. doi: 10.3389/fmicb.2022.905050 (PMC9244563; doi:10.3389/fmicb.2022.905050)
Supplement: Supplementary Table 1 — Ingredient and nutrient composition of basal diets (common for all three trials). [file Table_1.docx]

**Supplementary Table 1.** Ingredient and nutrient composition on basal Diet (common for all three trials)

| Ingredients Composition (g/kg) | Pre-Starter  (d 1 - 14) | Starter  (d 15 - 28) | Finisher  (29 d onwards) |
| --- | --- | --- | --- |
| Maize | 539.8 | 556.1 | 582.0 |
| Soy bean meal | 348.0 | 316.7 | 270.8 |
| Meat and bone meal | 39.7 | 39.7 | 39.7 |
| Stone grit | 24.5 | 10.6 | 10.7 |
| Fat (Vegetable oil) | 19.0 | 42.9 | 63.4 |
| Dicalcium phosphate (DCP) | 15.2 | 18.6 | 16.0 |
| DL-methionine | 3.05 | 2.65 | 2.67 |
| L- Lysine HCl | 1.35 | 0.750 | 1.12 |
| L-Threonine | 0.653 | 0.260 | 0.39 |
| Common salt | 4.0 | 4.1 | 4.0 |
| Trace Mineral Mix ^1^ | 1.225 | 2.0 | 2.5 |
| AB2D3K ^2^ | 0.204 | 0.360 | 0.375 |
| B complex vitamin ^3^ | 0.204 | 0.360 | 0.375 |
| Choline chloride | 1.02 | 2.0 | 2.5 |
| Toxin binder (Bentonite) | 1.02 | 2.0 | 2.5 |
| Sodium bicarbonate | 1.02 | 1.0 | 1.0 |
| Nutrient Composition (%) | | | |
| Metabolizable energy (Kcal/kg) ^4^ | 3000 | 3100 | 3250 |
| Protein ^5^ | 23.7 | 22.3 | 23.7 |
| d Lysine^4^ | 1.28 | 1.15 | 1.06 |
| d Methionine^4^ | 0.64 | 0.57 | 0.56 |
| d TSAA^4^ | 0.95 | 0.87 | 0.83 |
| d Threonine^4^ | 0.86 | 0.77 | 0.71 |
| d Tryptophan^4^ | 0.27 | 0.25 | 0.23 |
| Calcium^5^ | 0.96 | 0.87 | 0.88 |
| Total Phosphorus^5^ | 0.75 | 0.70 | 0.68 |
| Available Phosphorus^4^ | 0.48 | 0.44 | 0.44 |

1 composition (g/kg): manganese:110; zinc:100; copper:20; iron:110; selenium:1.5; iodine: 2.5; cobalt:1.0; chromium: 0.4;

2 composition (per g): retinolacetate: 82500IU; riboflavin: 50mg; cholecalciferol: 12000IU; menadione: 12000IU;

3 composition (mg/per g): thiamin:4; pyridoxin:8; cyanocobalamine:0.04; α-tocopherol:40; Calcium D Pantothenate:40; Niacin: 60; biotin:1.2;folic acid:4

4 Calculated

5 Analysed
