# Supplementary material for: Effects of Dietary Antimicrobial Growth Promoters on Performance Parameters and Abundance and Diversity of Broiler Chicken Gut Microbiome and Selection of Antibiotic Resistance Genes
Source: Front Microbiol. 2022 Jun 16;13:905050. doi: 10.3389/fmicb.2022.905050 (PMC9244563; doi:10.3389/fmicb.2022.905050)
Supplement: Supplementary Table 2 — Meat processing parameters during cycle 3 in different groups. [file Table_2.docx]

| **Treatment**  **AGP g/tonne** | **Live wt** | **RTC** | **Breast** | **Liver** | **Fat** | **Gizzard** | **Spleen** | **Heart** | **Bursa** | **Giblet** |
| --- | --- | --- | --- | --- | --- | --- | --- | --- | --- | --- |
| 1 (control) | 1880 | 1496 | 440.7 | 38.12 | 24.42 | 38.17 | 2.400 | 10.533 | 3.150 | 86.82 |
| 2 (Virginiamycin) 40g/tonne | 1993 | 1547 | 476.0 | 36.48 | 28.12 | 36.17 | 2.683 | 9.117 | 3.033 | 81.77 |
| 3 (CTC) 330g/tonne | 2012 | 1599 | 504.2 | 38.62 | 23.30 | 32.55 | 2.533 | 9.417 | 2.283 | 80.58 |
| 4 (BMD) 500g/tonne | 2006 | 1592 | 476.5 | 35.78 | 33.12 | 37.92 | 2.750 | 10.533 | 2.550 | 84.23 |
| 5 (Lincomycin) 40g/tone | 2016 | 1593 | 528.8 | 35.95 | 23.94 | 33.38 | 2.217 | 9.517 | 2.900 | 78.85 |
| 6 (Tylosin) 500g/tonne | 1936 | 1503 | 461.4 | 34.88 | 21.85 | 35.38 | 2.100 | 9.717 | 2.283 | 79.98 |
| SEM | 34.31 | 29.77 | 12.27 | 0.749 | 1.293 | 1.109 | 0.154 | 0.332 | 0.211 | 1.705 |
| P value | 0.855 | 0.860 | 0.384 | 0.714 | 0.115 | 0.637 | 0.828 | 0.775 | 0.772 | 0.796 |

**Supplementary Table 2.** Meat processing parameters during cycle 3 in different groups
