# Supplementary material for: Effects of Dietary Antimicrobial Growth Promoters on Performance Parameters and Abundance and Diversity of Broiler Chicken Gut Microbiome and Selection of Antibiotic Resistance Genes
Source: Front Microbiol. 2022 Jun 16;13:905050. doi: 10.3389/fmicb.2022.905050 (PMC9244563; doi:10.3389/fmicb.2022.905050)
Supplement: Supplementary Table 3 — Effect of supplementing antimicrobial growth promoters on apparent total tract digestibility (nutrient retention) during experimental cycle 1. [file Table_3.docx]

**Supplementary Table 3.** Effect of supplementing antimicrobial growth promoters on apparent total tract digestibility (nutrient retention) during experimental cycle 1

|  | **Total tract nutrient retention (g /kg)** | | |
| --- | --- | --- | --- |
|  | **DM** | **EE** | **CP** |
|  | Cycle 1 | Cycle 1 | Cycle 1 |
| Control | 78.15 | 91.77 | 88.27 |
| V | 78.29 | 92.41 | 89.84 |
| CT | 76.20 | 91.58 | 88.49 |
| B | 79.59 | 92.84 | 89.27 |
| L | 79.74 | 92.99 | 88.85 |
| T | 81.06 | 93.66 | 90.18 |
| P value | 0.601 | 0.302 | 0.735 |
| N | 4 | 4 | 4 |
| SEM | 1.945 | 0.683 | 1.016 |

V, virginiamycin; CT, chloro tetra cycline; B, bacitracin methylene di salicylate; L, lincomycin; T, Tylosin; P, probability; N, number of replicates; SEM, standard error mean
