# Supplementary material for: Effects of Dietary Antimicrobial Growth Promoters on Performance Parameters and Abundance and Diversity of Broiler Chicken Gut Microbiome and Selection of Antibiotic Resistance Genes
Source: Front Microbiol. 2022 Jun 16;13:905050. doi: 10.3389/fmicb.2022.905050 (PMC9244563; doi:10.3389/fmicb.2022.905050)
Supplement: Supplementary Table 4 — Statistical analysis of alpha diversity measures for comparing different groups. [file Table_4.docx]

**Supplementary Table 4.** Statistical analysis of alpha diversity measures for comparing different groups

| **Metrices** | **Mean ± SD values#**  **C B CT L T V** | | | | | | **Krushkal Wallis test (p-values)** |
| --- | --- | --- | --- | --- | --- | --- | --- |
| Richness | | | | | | | |
| Observed richness | 13585±627 | 13284±1540 | 13535±1161 | 13929±1011 | 13197±1377 | 13439±2157 | 0.976 |
| Chao1 | 14721±623 | 14275±1469 | 14251±1301 | 14648±974 | 14065±1255 | 14361±2067 | 0.931 |
| ACE | 14639±603 | 14169±1435 | 14199±1281 | 14589±959 | 13999±1257 | 14305±2036 | 0.951 |
| Diversity | | | | | | | |
| Simpson | 0.942±0.039 | 0.959±0.025 | 0.974±0.002 | 0.970±0.053 | 0.963±0.015 | 0.955±0.033 | 0.293 |
| Shannon | 4.57±0.479 | 4.85±0.519 | 5.18±0.103 | 5.16±0.229 | 4.91±0.447 | 4.82±0.653 | 0.295 |
| fisher | 1592±83 | 1553±203 | 1586±154 | 1638±134 | 1541±183 | 1575±282 | 0.976 |

# data were rarefied to the minimum library size;
