# Supplementary material for: Effects of Dietary Antimicrobial Growth Promoters on Performance Parameters and Abundance and Diversity of Broiler Chicken Gut Microbiome and Selection of Antibiotic Resistance Genes
Source: Front Microbiol. 2022 Jun 16;13:905050. doi: 10.3389/fmicb.2022.905050 (PMC9244563; doi:10.3389/fmicb.2022.905050)
Supplement: Supplementary Table 5 — List of antimicrobial resistance genes detected in shotgun sequencing data. [file Table_5.docx]

**Supplementary Table 5.** List of antimicrobial resistance genes detected in shotgun sequencing data

| **Antimicrobial name/category** | **Antimicrobial resistance Genes detected** |
| --- | --- |
| Aminoglycoside | Ant, aad, aph, aac,baes, kdpE,sat4A, AGly,aadA, APH |
| Lincomycin | Lnu A, lnu C |
| Beta lactum | blaTEM, TEM,cblA,cfxA2, blaCTX-M, blaSHV,blaOXA-1 |
| Tetracycline | Tet, tet 32, tet (O) |
| Peptide | bacA,arnA |
| Macrolide-lincosamide-streptograminB(MLS) | Erm, LnuB, ermB,MsrE, vatE |
| Sulfonamide | sul |
| Trimethoprim | dfrF |
| Fluoroquinolone | Qnr, PAT A |
| Nucleoside | SAT4, SAT2 |
| Multiple antibiotic(multidrug) | Emr K, emr R, emr Y, ade J, adeN,msbA,mdtG,mar A, kdpE,kdpD,gad X, evg S, evg A, PmrF , PmrC, acr F, Isa E, tol C, mar A, ade K, adeC, mdtE, mdt n, bacS, pat A |
| StreptograminA | VatE |
| Chloramphenicol | cmlA |
| Metronidazole | nimE |
