# Supplementary material for: Effects of Dietary Antimicrobial Growth Promoters on Performance Parameters and Abundance and Diversity of Broiler Chicken Gut Microbiome and Selection of Antibiotic Resistance Genes
Source: Front Microbiol. 2022 Jun 16;13:905050. doi: 10.3389/fmicb.2022.905050 (PMC9244563; doi:10.3389/fmicb.2022.905050)
Supplement: Supplementary Table 6 — Effects of different antimicrobial growth promoters (AGPs) on the prevalence of antimicrobial resistance genes (ARGs) as determined by conventional PCR and qPCR. [file Table_6.docx]

**Supplementary Table 6.** Effects of different antimicrobial growth promoters (AGPs) on the prevalence of antimicrobial resistance genes (ARGs) as determined by conventional PCR and qPCR

| ARG types | ARGs (subtypes of ARGs) | Control | | Virginamycin | | CTC | | BMD | | Lincomycin | | Tylosin | |
| --- | --- | --- | --- | --- | --- | --- | --- | --- | --- | --- | --- | --- | --- |
|  |  | Cycle1 | Cycle3 | Cycle1 | Cycle3 | Cycle1 | Cycle3 | Cycle1 | Cycle3 | Cycle1 | Cycle3 | Cycle1 | Cycle3 |
| Conventional PCR (% occurence) | | | | | | | | | | | | | |
| Beta lactamase/ESBL | *blaTEM* | 16.6 | 16.6 | ND | 16.6 | 33.3 | 50 | ND | 33.3 | 16.6 | ND | 33.3 | 33.3 |
|  | *blaOXA/SHV/NDM* | ND | ND | ND | ND | ND | ND | ND | ND | ND | ND | ND | ND |
| ESBL | *bla CTX-M gr 1/2/9/(8/25)* | ND | ND | ND | ND | ND | ND | ND | ND | ND | ND | ND | ND |
|  | *Bla CTX-M(U)* | ND | 16.6 | ND | ND | ND | ND | ND | ND | ND | ND | ND | ND |
| colistin | *mcr 1/2/3/4/5* | ND | ND | ND | ND | ND | ND | ND | ND | ND | ND | ND | ND |
| quinolone | *qnrA* | 33.3 | 33.3 | ND | ND | 16.6 | ND | ND | ND | 33.3 | ND | 33.3 | ND |
|  | *qnrB* | ND | 16.6 | ND | 16.6 | 16.6 | 33.3 | ND | 33.3 | ND | ND | 50 | 16.6 |
| quinolone | *qepA* | 66.6 | 66.6 | 83.3 | 83.3 | 33.3 | 33.3 | ND | ND | 50 | 50 | ND | ND |
| MDR (efflux pump) | *oqxAB* | ND | ND | ND | ND | ND | ND | ND | ND | ND | ND | ND | ND |
| qPCR ( range of copy no/50ng DNA in samples detected positive in conventional PCR ) | | | | | | | | | | | | | |
| Beta lactamase/ESBL | *blaTEM* | 0.183 | 0.444 | ND | 0.617 | 0.036 | 0.128-2.02 | ND | 0.239-0.264 | 0.123 | ND | 0.023-0.027 | 0.023-0.027 |
| ESBL | *Bla CTXM(U)* | ND | 0.030 | ND | ND | ND | ND | ND | ND | ND | ND | ND | ND |
| quinolone | *qnrB* | ND | 0.005 | ND | 0.048 | ND | 0.003-0.024 | ND | 0.001-0.002 | ND | ND | 0.017-0.133 | ND |

ESBL, Extended spectrum beta lactamase ( 3^rd^ gen cephalosporin /aztreonam); ND, Not detected
