# Supplementary material for: Effects of Dietary Antimicrobial Growth Promoters on Performance Parameters and Abundance and Diversity of Broiler Chicken Gut Microbiome and Selection of Antibiotic Resistance Genes
Source: Front Microbiol. 2022 Jun 16;13:905050. doi: 10.3389/fmicb.2022.905050 (PMC9244563; doi:10.3389/fmicb.2022.905050)
Supplement: Supplementary Data Sheet 1 — Details of PCR and qPCR protocols used for the detection of antimicrobial resistance genes in metagenomic DNA isolated from gut content. [file Data_Sheet_1.docx]

**Supplementary Data Sheet 1.** Detail of PCR and qPCR protocols used for the detection of antimicrobial resistance genes in metagenomic DNA isolated from gut content

1. **Screening of antibiotic resistance genes (*bla, mcr* and quinolone resistance) by multiplex/simplex PCR respectively:**

**Multiplex PCR for *bla* genes *(Dallenne et al., 2010)*:**

| **Gene** | **Primer sequence** | | **Amplicon size (bp)** |
| --- | --- | --- | --- |
| ***bla_TEM_*** | **FP** | CATTTCCGTGTCGCCCTTATTC | **800** |
|  | **RP** | CGTTCATCCATAGTTGCCTGAC |  |
| ***bla_SHV_*** | **FP** | AGCCGCTTGAGCAAATTAAAC | **713** |
|  | **RP** | ATCCCGCAGATAAATCACCAC |  |
| ***bla_OXA_*** | **FP** | GGCACCAGATTCAACTTTCAAG | **564** |
|  | **RP** | GACCCCAAGTTTCCTGTAAGTG |  |
| ***bla_NDM_*** | **FP** | TGGATCAAGCAGGAGATCAA | **250** |
|  | **RP** | GGCCGGGGTAAAATACCTT |  |

- Use the above cPCR primers for screening the test samples (individual isolates) for *bla* genes
- Prepare the reaction mixture for 19 μL total volume per tube without the addition of DNA template. Each run should include positive controls and a non-template control (NTC). For NTC use NFW instead of template
- The guideline for preparation of reaction mixture (20 μL) as per the Takara manual is as follows:

| **Contents** | **Volume/Final concentration** |
| --- | --- |
| ******EmeraldAmp GT PCR Master Mix (2X Premix) | 10 μL |
| Template | < 500 ng |
| Forward Primer | 0.2 μM (final conc.) |
| Reverse Primer | 0.2 μM (final conc.) |
| Sterile nuclease-free water | up to 20 μL |
| Final Volume of reaction mixture | 20 μL |

**** EmeraldAmp GT PCR Master Mix** is a double strength premix composed of a DNA polymerase, optimized reaction buffer, dNTPs, a density reagent and a vivid green dye that will separate into blue and yellow dyefronts when run on an agarose gel.

| **Contents** | | **Volume for 1 reaction (20 μl)** |
| --- | --- | --- |
| Mastermix | | 10 |
| TEM | FP | 0.5 |
|  | RP | 0.5 |
| SHV | FP | 0.5 |
|  | RP | 0.5 |
| OXA | FP | 0.5 |
|  | RP | 0.5 |
| NDM | FP | 0.5 |
|  | RP | 0.5 |
| NFW | | 5 |
| **Total volume** | | **19** |

- An example of reaction mixture to be prepared is as follows:
- Add 1 μL DNA template to the **19 μL** reaction mixture for a total volume of 20 μL
- Run the reaction as follows for multiplex PCR for the *bla* genes

95 ℃/ 12 mins

95 ℃/ 30 s

55 ℃/ 30 s x **30 cycles**

72 ℃/ 1 min

72 ℃/ 7 min

4 ℃/ ∞

**Simplex PCR for *bla_CTX-M_* genes (Initial screening with CTX-M universal primer *(Boyd et al., 2004)* :**

| **Gene** | **Primer sequence** | | **Amplicon size (bp)** |
| --- | --- | --- | --- |
| ***bla_CTX-M_ (Universal)*** | **FP** | ATGTGCAGCACCAGTAAAGTGATGGC | **593** |
|  | **RP** | TGGGTAAAGTAAGTCACCAGAATCAGCGG |  |

- Perform simplex PCR for screening the of CTX-M genes with CTX-M universal primer first.
- Only if an isolate is found to be positive for amplification with the CTX-M universal primer, proceed with the multiplex PCR for the CTX-M genes.
- Prepare the reaction mixture for 19.5 μL total volume/ tube without the addition of DNA template. The run of each PCR should include positive controls and a non-template control (NTC)

|  | | **Volume for 1 reaction (20 μL)** |
| --- | --- | --- |
| Mastermix | | 10 |
| CTX-M | FP | 0.5 |
|  | RP | 0.5 |
| NFW | | 8 |
| **Total volume** | | **19** |

- Add 1 μL of DNA template to the **19 μL** of reaction mixture to make a total volume of 20 μL
- Run the reaction as follows for simplex PCR for the *CTX-M* genes

95 ℃/ 12 mins

95 ℃/ 30 s

55 ℃/ 30 s x **30 cycles**

72 ℃/ 1 min

72 ℃/ 7 min

4 ℃/ ∞

***Multiplex PCR for bla_CTX-M_* genes:**

- Once an isolate is positive for amplification with the CTX-M universal primer, it needs to be screened for the different CTX-M groups by multiplex PCR ***(Dallenne et al., 2010).***
- Following are the different CTX-M group primers to be used along with their respective amplicon sizes:

| **Gene** | **Primer sequence** | | **Amplicon size (bp)** |
| --- | --- | --- | --- |
| ***bla_CTX-M_* group 1**  **(CTX-M 1, 3, 15)** | **FP** | TTAGGAAGTGTGCCGCTGTA | **688** |
|  | **RP** | CGATATCGTTGGTGGTGCCAT |  |
| ***bla_CTX-M_* group 2**  **(CTX-M 2)** | **FP** | CGTTAACGGCACGATGAC | **404** |
|  | **RP** | CGATATCGTTGGTGGTGCCAT |  |
| ***bla_CTX-M_* group 9**  **(CTX-M 9, 14)** | **FP** | TCAAGCCTGCCGATCTGGT | **561** |
|  | **RP** | TGATTCTCGCCGCTGAAG |  |
| ***bla_CTX-M_* group 8/25**  **(CTX-M 8, 25, 26, 39-41)** | **FP** | AACACGCAGACGCTCTAC | **326** |
|  | **RP** | TCGAGCCGGAACGTGTCAT |  |

- Use the above cPCR primers for screening the samples (individual isolates that were positive for amplification with the CTX-M universal primer) for *bla* genes
- Prepare the reaction mixture for 19 μL total volume per tube without the addition of DNA template. The run of each PCR should include positive controls and a non-template control (NTC)
- An example of the reaction mixture to be prepared is as follows:

| **Contents** | | **Volume for 1 reaction (20 μl)** |
| --- | --- | --- |
| Mastermix | | 10 |
| ***bla_CTX-M_* group 1** | FP | 0.5 |
|  | RP | 0.5 |
| ***bla_CTX-M_* group 2** | FP | 0.5 |
|  | RP | 0.5 |
| ***bla_CTX-M_* group 9** | FP | 0.5 |
|  | RP | 0.5 |
| ***bla_CTX-M_* group 8/25** | FP | 0.5 |
|  | RP | 0.5 |
| NFW | | 5 |
| **Total volume** | | **19** |

- Add 1 μL of DNA template to the **19 μL** of reaction mixture for total volume of 20 μL
- Run the reaction as follows for multiplex PCR for the *CTX-M* genes

95 ℃/ 12 mins

95 ℃/ 30 s

55 ℃/ 30 s x **30 cycles**

72 ℃/ 1 min

72 ℃/ 7 min

4 ℃/ ∞

***Multiplex PCR for Quinolone resistance* genes *(Ciesielczuk et al. 2013):***

Perform the screening of quinolone resistance genes by multiplex PCR in 2 sets based on the amplicon sizes of the gene primers

| **Gene** | **Primer sequence (Set 1)** | | **Amplicon size (bp)** |
| --- | --- | --- | --- |
| ***qnrA*** | **FP** | CAGCAAGAGGATTTCTCACG | 631 |
|  | **RP** | AATCCGGCAGCACTATTACTC |  |
| ***qnrB*** | **FP** | GGCTGTCAGTTCTATGATCG | 489 |
|  | **RP** | GAGCAACGATGCCTGGTAG |  |
| ***oqxAB*** | **FP** | CCGCACCGATAAATTAGTCC | 313 |
|  | **RP** | GGCGAGGTTTTGATAGTGGA |  |
| ***qepA*** | **FP** | GCAGGTCCAGCAGCGGGTAG | 218 |
|  | **RP** | CTTCCTGCCCGAGTATCGTG |  |

| **Gene** | **Primer sequence (Set 2)** | | **Amplicon size (bp)** |
| --- | --- | --- | --- |
| ***qnrD*** | **FP** | CGAGATCAATTTACGGGGAATA | 582 |
|  | **RP** | AACAAGCTGAAGCGCCTG |  |
| ***qnrS*** | **FP** | GCAAGTTCATTGAACAGGGT | 427 |
|  | **RP** | TCTAAACCGTCGAGTTCGGCG |  |
| ***aac(6’)-Ib-cr*** | **FP** | TTGGAAGCGGGGACGGAC | 260 |
|  | **RP** | ACACGGCTGGACCATA |  |
| ***qnrC*** | **FP** | GCAGAATTCAGGGGTGTGAT | 118 |
|  | **RP** | AACTGCTCCAAAAGCTGCTC |  |

- Use the above cPCR primers for screening each sample (individual isolates or pooled samples) for *quinolone resistance* genes in two different sets.
- Prepare the reaction mixture for 19 μL total volume per tube without the addition of DNA template. Each run should include positive controls and a non-template control (NTC). For NTC use NFW instead of template
- An example of reaction mixture to be prepared is as follows:

| **Contents (Set 1)** | | **Volume for 1 reaction (20 μL)** | **Contents (Set 2)** | | **Volume for 1 reaction (20 μL)** |
| --- | --- | --- | --- | --- | --- |
| Mastermix | | 10 | Mastermix | | 10 |
| ***qnrA*** | FP | 0.5 | ***qnrD*** | FP | 0.5 |
|  | RP | 0.5 |  | RP | 0.5 |
| ***qnrB*** | FP | 0.5 | ***qnrS*** | FP | 0.5 |
|  | RP | 0.5 |  | RP | 0.5 |
| ***oqxAB*** | FP | 0.5 | ***aac(6’)-Ib-cr*** | FP | 0.5 |
|  | RP | 0.5 |  | RP | 0.5 |
| ***qepA*** | FP | 0.5 | ***qnrC*** | FP | 0.5 |
|  | RP | 0.5 |  | RP | 0.5 |
| NFW | | 5 | NFW | | 5 |
| **Total volume** | | **19** | **Total volume** | | **19** |

- Add 1 μL of DNA Add template to the **19 μL** of reaction mixture to make a total volume of 20 μL

Run the reaction for multiplex PCR program for **quninolone resistance** genes as follows:

95 ℃/ 15 mins

94 ℃/ 30 s

55 ℃/ 90 s x **30 cycles**

72 ℃/ 90 s

72 ℃/ 10 min

4 ℃/ ∞

***Multiplex PCR for mcr* genes *(Lescat, Poirel, and Nordmann 2018)*:**

| **Gene** | **Primer sequence** | | **Amplicon size (bp)** |
| --- | --- | --- | --- |
| ***mcr 1*** | **FP** | ATGCCAGTTTCTTTCGCGTG | **502** |
|  | **RP** | TCGGCAAATTGCGCTTTTGGC |  |
| ***mcr 2*** | **FP** | GATGGCGGTCTATCCTGTAT | **379** |
|  | **RP** | AAGGCTGACACCCCATGTCAT |  |
| ***mcr 3*** | **FP** | ACCAGTAAATCTGGTGGCGT | **296** |
|  | **RP** | AGGACAACCTCGTCATAGCA |  |
| ***mcr 4*** | **FP** | TTGCAGACGCCCATGGAATA | **207** |
|  | **RP** | GCCGCATGAGCTAGTATCGT |  |
| ***mcr 5*** | **FP** | GGACGCGACTCCCTAACTTC | **608** |
|  | **RP** | ACAACCAGTACGAGAGCACG |  |

- Use the above cPCR primers for screening the test samples (individual isolates or pooled samples) for *mcr* genes
- Prepare the reaction mixture for 19 μL total volume per tube without the addition of DNA template. Each run should include positive controls and a non-template control (NTC). For NTC use NFW instead of template
- An example of reaction mixture to be prepared is as follows:

| **Contents** | | **Volume for 1 reaction (20 μl)** |
| --- | --- | --- |
| Mastermix | | 10 |
| ***mcr 1*** | FP | 1 |
|  | RP | 1 |
| ***mcr 2*** | FP | 1 |
|  | RP | 1 |
| ***mcr 3*** | FP | 1 |
|  | RP | 1 |
| ***mcr 4*** | FP | 1 |
|  | RP | 1 |
| ***mcr 5*** | FP | 1 |
|  | RP | 1 |
| NFW | | 0 |
| **Total volume** | | **19** |

- Add 1 μL DNA template to the **19 μl** reaction mixture for a total volume of 20 μL
- Run the reaction for ***mcr*** genes as follows:

94 ℃/ 4 min

94 ℃/ 5 s

59 ℃/ 20 s x **30 cycles**

72 ℃/ 5 min

4 ℃/ ∞

1. **qPCR analysis**

All the samples that are identified for the respective AMR gene amplification by conventional PCR are detected for their copy numbers by qPCR analysis (Quant Studio 5, Applied Biosystems).

Every reaction contains one set of housekeeping gene (16s) and one known gene control in triplicates respectively. Copy numbers for each of these two genes are set from 10^10^ - 10^5^ (in Thermofisher Scientific Dilution Calculator) and subsequently used for obtaining a standard curve post amplification. A logarithmic standard graph is plotted in the form of C_t_ vs Copy number.

In addition, the test samples for detection of copy number of the respective AMR gene and NTC are set in duplicates. The copy numbers aree detected by placing the C_t_ values of these test samples in the standard curve.

The samples are prepared for 10 μL volume reaction mixture

The guidelines for preparation of reaction mixture (10 μL) as per the Applied Biosystems™ manual (Power SYBR™ Green PCR Master Mix- ROX dye based, Cat no. 4367659) are as follows:

| **Contents** | **Volume/Final concentration** |
| --- | --- |
| Power SYBR™ Green PCR Master Mix (2X Premix) | 5 μL (1X) |
| Template | 1-100 ng |
| Forward Primer | 50-100 nM (used 500 nM) |
| Reverse Primer | 50-100 nM (used 500 nM) |
| Sterile nuclease-free water | up to 10 μL |
| Final Volume of reaction mixture | 10 μL |

Prepare the reaction mixture for 9.5 μL total volume/ tube without the addition of DNA template. The run of each PCR should include positive controls and a non-template control (NTC)

|  | | **Volume for 1 reaction (20 μL)** |
| --- | --- | --- |
| Mastermix (2X) | | 10 |
| AMR gene primer | **FP** | 0.5 |
|  | **RP** | 0.5 |
| NFW | | 8 |
| **Total volume** | | **19** |

Add 0.5 μL of DNA template to the **9.5 μL** of reaction mixture to make a total volume of 10 μL

**qPCR primers for 16s gene are as follows (*Suzuki et al., 2000*):**

| **Gene** | **Primer sequence (qPCR)** | | **Amplicon size (bp)** |
| --- | --- | --- | --- |
| ***16s*** | **FP** | CGGTGAATACGTTCTCGG | 142 |
|  | **RP** | GGATACCTTGTTACGACT |  |

**qPCR primers for *blaI* and bla*_CTX-M_* genes are as follows *(Singh, Pfeifer, and Mustapha 2016)*:**

| **Gene** | **Primer sequence (qPCR)** | | **Amplicon size (bp)** |
| --- | --- | --- | --- |
| ***bla_TEM_*** | **FP** | GATACGGGAGGGCTTACCAT | 146 |
|  | **RP** | GGATGGAGGCGGATAAAGTT |  |
| ***bla_SHV_*** | **FP** | GGTCAGCGAAAAACATCTTG | 195 |
|  | **RP** | GCCTCATTCAGTTCCGTTTC |  |
| ***bla_OXA_*** | **FP** | AGCAAAGGAATGGCAAGAAA | 65 |
|  | **RP** | CGCCCTGTGATTTATGTTCA |  |
| ***bla_NDM_*** | **FP** | TGGATCAAGCAGGAGATCAA | 250 |
|  | **RP** | GGCCGGGGTAAAATACCTT |  |
| ***bla_CTX-M_*** | **FP** | AATCTGACGCTGGGTAAAG | 140 |
|  | **RP** | CCGCTGCCGGTTTTATC |  |

**The qPCR program for *blaI* (TEM, SHV, OXA, NDM) and *bla_CTX-M_* genes are as follows:**

95 ℃/ 12 mins

95 ℃/ 15 s

60 ℃/ 30 s x **40 cycles**

72 ℃/ 20 s

4 ℃/ ∞

**qPCR primers for *quinolone resistance* (*qnrA, qnrB and qnrS*) genes are as follows *(Marti and Balcázar 2013)*:**

| **Gene** | **Primer sequence (qPCR)** | | **Amplicon size (bp)** |
| --- | --- | --- | --- |
| ***qnrA*** | **FP** | CAGCAAGAGGATTTCTCACG | 631 |
|  | **RP** | AATCCGGCAGCACTATTACTC |  |
| ***qnrB*** | **FP** | GGCTGTCAGTTCTATGATCG | 489 |
|  | **RP** | GAGCAACGATGCCTGGTAG |  |
| ***qnrS*** | **FP** | GCAAGTTCATTGAACAGGGT | 427 |
|  | **RP** | TCTAAACCGTCGAGTTCGGCG |  |

**The qPCR program for *quinolone resistance* (*qnrA, qnrB and qnrS*) genes are as follows:**

qPCR program for *qnrA*:

95 ℃/ 12 mins

95 ℃/ 15 s

57 ℃/ 20 s x **40 cycles**

72 ℃/ 1 min

4 ℃/ ∞

qPCR program for *qnrB* and *qnrS*:

95 ℃/ 12 mins

95 ℃/ 15 s

62 ℃/ 20 s x **40 cycles**

72 ℃/ 1 min

4 ℃/ ∞

**NOTE:** qPCR for colistin resistance genes (*mcr1-5*) was not performed as there was no amplification of these genes when tried for amplification by conventional PCR.

**References**

- Boyd DA, Tyler S, Christianson S, McGeer A, Muller MP et al. 2004. “Complete Nucleotide Sequence of a 92-Kilobase Plasmid Harboring the CTX-M-15 Extended-Spectrum Beta-Lactamase Involved in an Outbreak in Long-Term-Care Facilities in Toronto, Canada.” Antimicrobial Agents and Chemotherapy 48 (10): 3758–64. https://doi.org/10.1128/AAC.48.10.3758-3764.2004.
- Ciesielczuk H, Hornsey M, Choi V, Woodford N, Wareham DW. 2013. “Development and Evaluation of a Multiplex PCR for Eight Plasmid-Mediated Quinolone-Resistance Determinants.” Journal of Medical Microbiology 62 (PART 12): 1823–27. https://doi.org/10.1099/jmm.0.064428-0.
- Dallenne C, Da Costa A, Decré D, Favier C, and Arlet G. 2010. “Development of a Set of Multiplex PCR Assays for the Detection of Genes Encoding Important β-Lactamases in *Enterobacteriaceae*.” Journal of Antimicrobial Chemotherapy 65 (3): 490–95. https://doi.org/10.1093/jac/dkp498.
- Lescat M, Poirel L, Nordmann P. 2018. “Rapid Multiplex Polymerase Chain Reaction for Detection of Mcr-1 to Mcr-5 Genes.” Diagnostic Microbiology and Infectious Disease 92 (4): 267–69. https://doi.org/10.1016/J.DIAGMICROBIO.2018.04.010.
- Marti E, Balcázar JL. 2013. “Real-Time PCR Assays for Quantification of Qnr Genes in Environmental Water Samples and Chicken Feces.” Applied and Environmental Microbiology 79 (5): 1743–45. https://doi.org/10.1128/AEM.03409-12.
- Singh P, Pfeifer Y, Mustapha A. 2016. “Multiplex Real-Time PCR Assay for the Detection of Extended-Spectrum β-Lactamase and Carbapenemase Genes Using Melting Curve Analysis.” Journal of Microbiological Methods 124 (May): 72–78. https://doi.org/10.1016/J.MIMET.2016.03.014.
- Suzuki MT., Taylor LT, DeLong EF. Quantitative analysis of small-subunit rRNA genes in mixed microbial populations via 5'-nuclease assays. *Appl Environ Microbiol*. 2000;66(11):4605-4614. doi:10.1128/aem.66.11.4605-4614.2000
