# Supplementary material for: Effects of Dietary Antimicrobial Growth Promoters on Performance Parameters and Abundance and Diversity of Broiler Chicken Gut Microbiome and Selection of Antibiotic Resistance Genes
Source: Front Microbiol. 2022 Jun 16;13:905050. doi: 10.3389/fmicb.2022.905050 (PMC9244563; doi:10.3389/fmicb.2022.905050)

## Slide 1
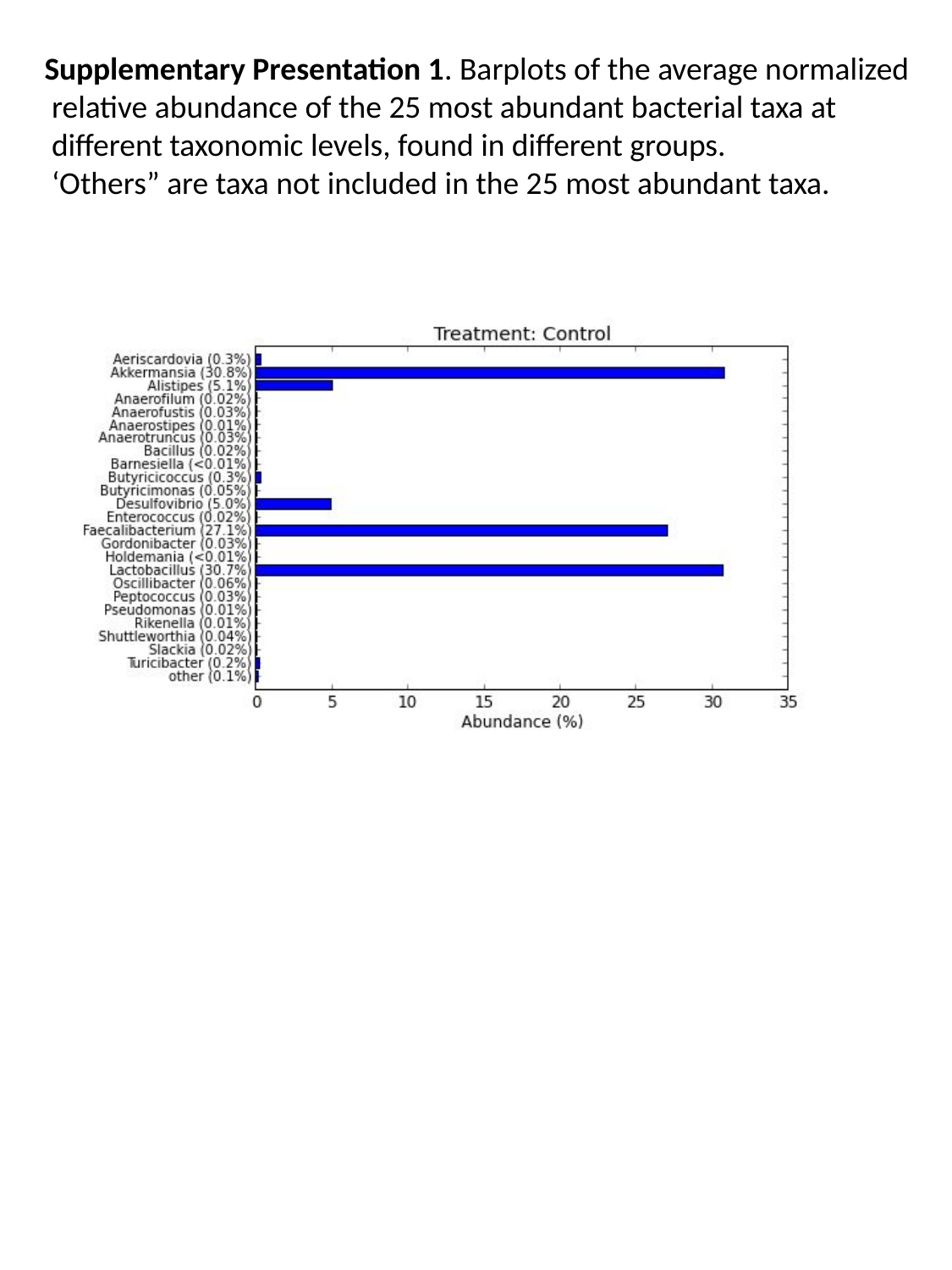

Supplementary Presentation 1. Barplots of the average normalized
 relative abundance of the 25 most abundant bacterial taxa at
 different taxonomic levels, found in different groups.
 ‘Others” are taxa not included in the 25 most abundant taxa.

## Slide 2
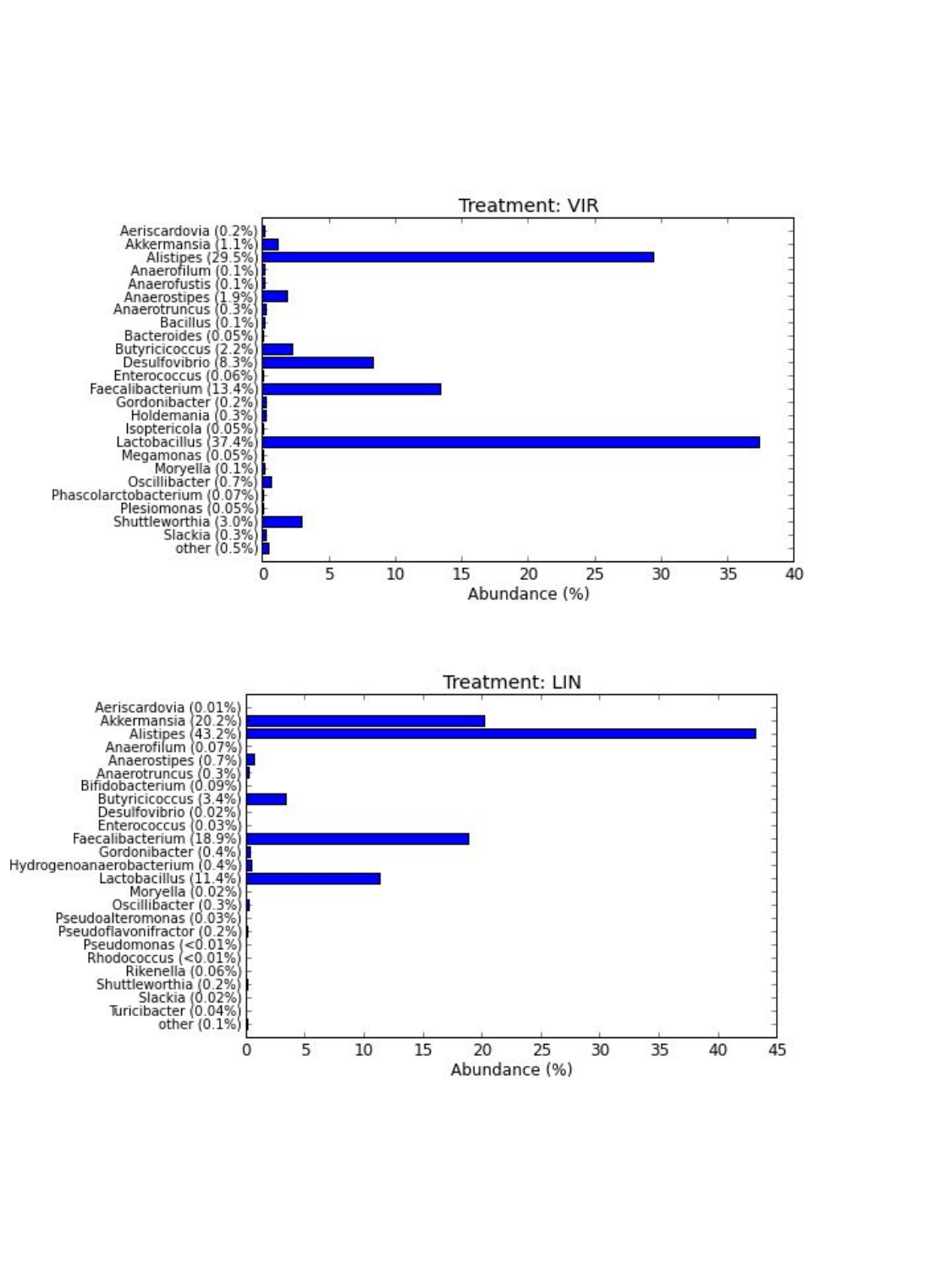

## Slide 3
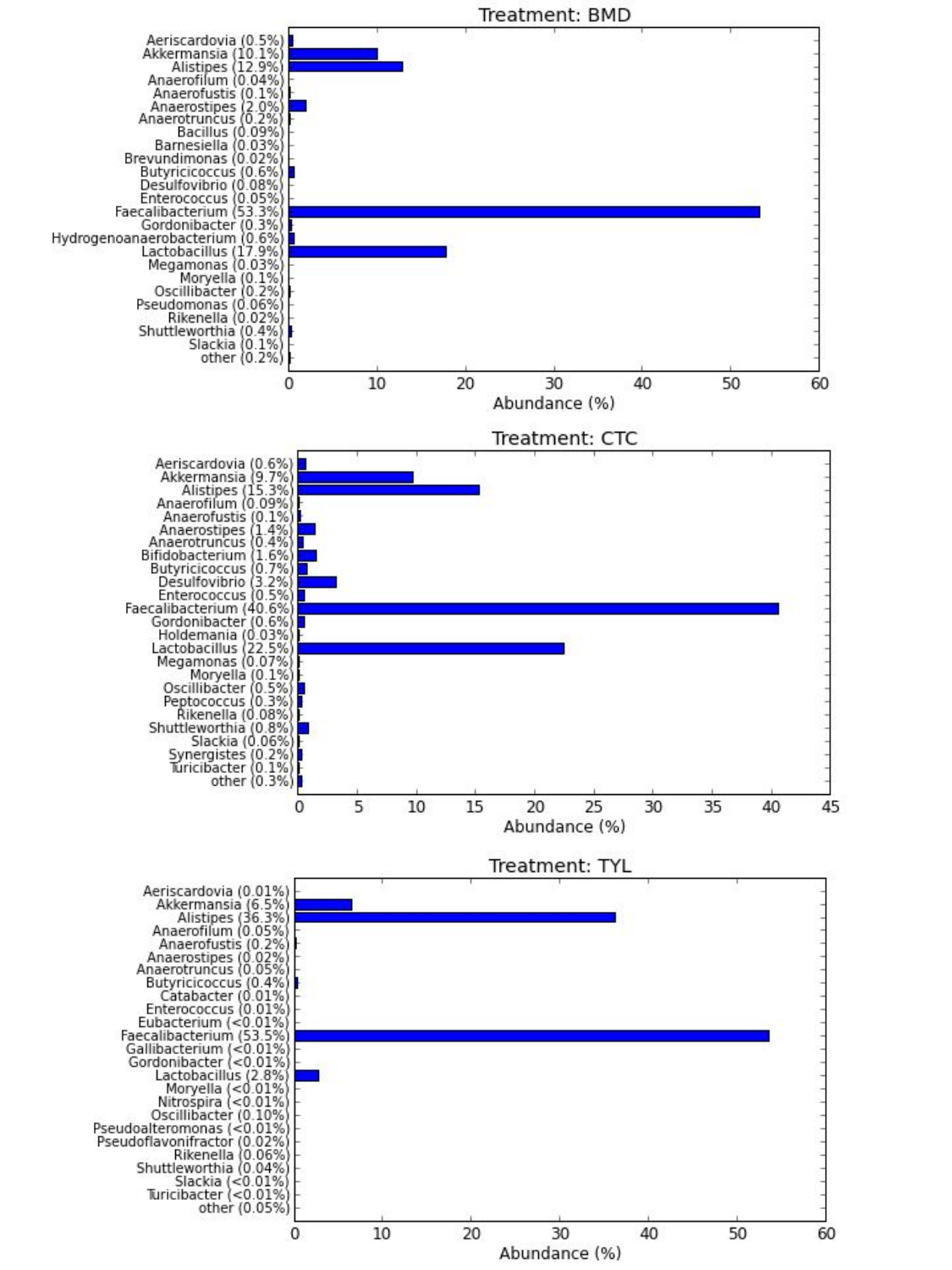

## Slide 4
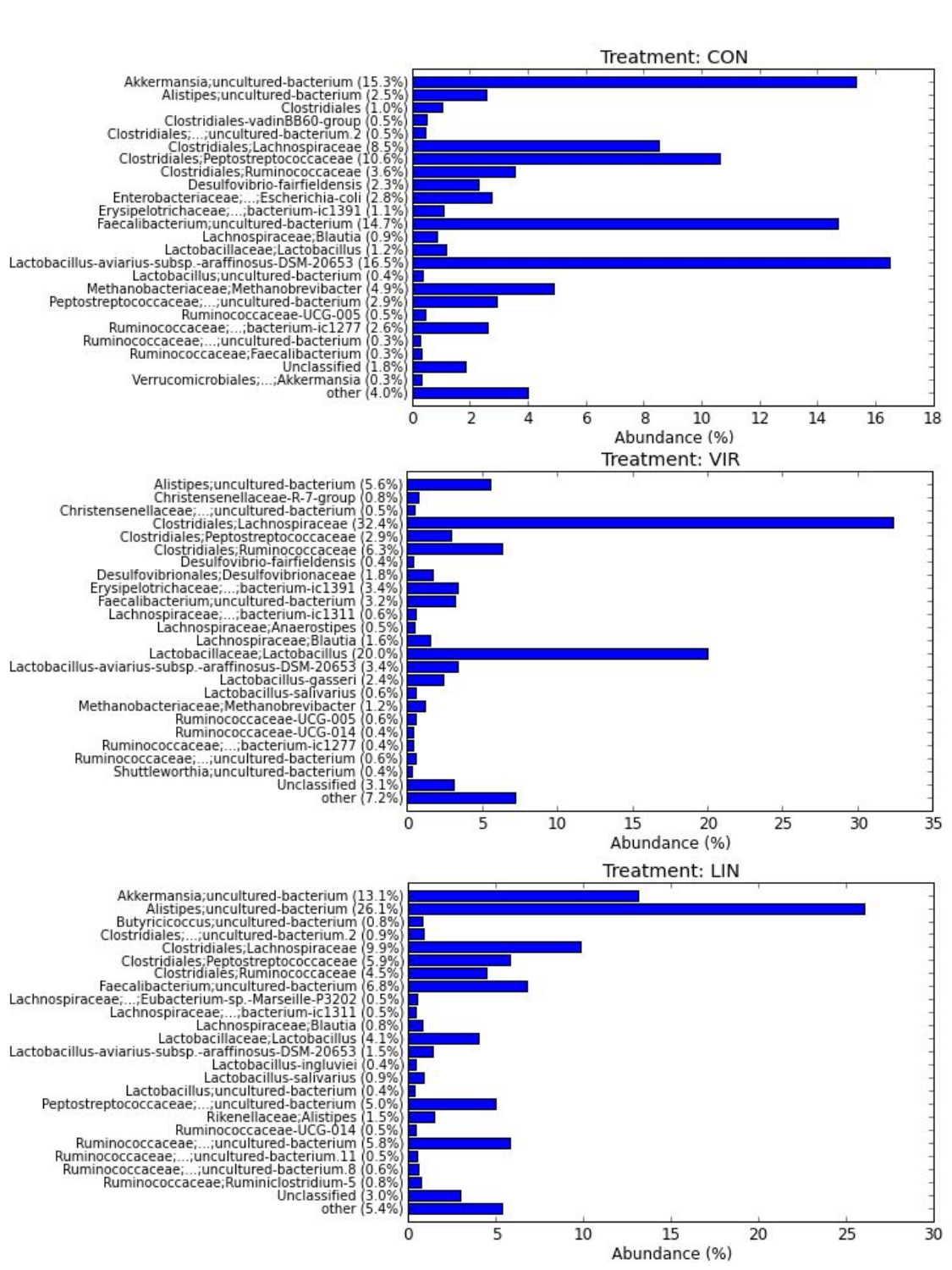

## Slide 5
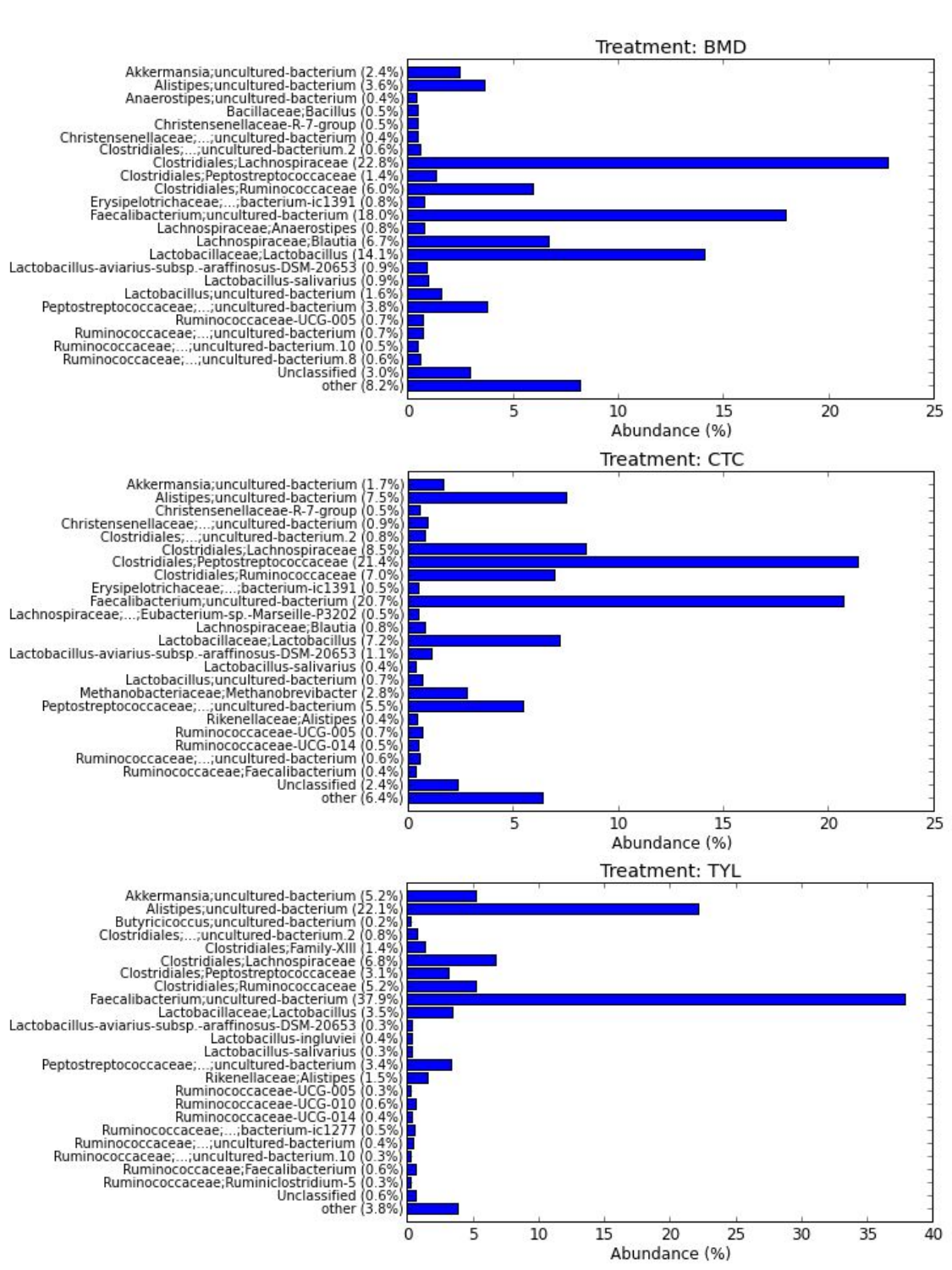

Supplement: Supplementary Presentation 1 — Barplots of the average normalized relative abundance of the 25 most abundant bacterial taxa at different taxonomic levels, found in different groups. “Others” are taxa not included in the 25 most abundant taxa. [file Presentation_1.pptx]
